# Interpersonal Agreement and Disagreement During Face-to-Face Dialogue: An fNIRS Investigation

**DOI:** 10.3389/fnhum.2020.606397

**Published:** 2021-01-13

**Authors:** Joy Hirsch, Mark Tiede, Xian Zhang, J. Adam Noah, Alexandre Salama-Manteau, Maurice Biriotti

**Affiliations:** ^1^Brain Function Laboratory, Department of Psychiatry, Yale School of Medicine, New Haven, CT, United States; ^2^Department of Neuroscience, Yale School of Medicine, New Haven, CT, United States; ^3^Department of Comparative Medicine, Yale School of Medicine, New Haven, CT, United States; ^4^Haskins Laboratories, New Haven, CT, United States; ^5^Department of Medical Physics and Biomedical Engineering, University College London, London, United Kingdom; ^6^Faculty of Arts and Humanities, University College London, London, United Kingdom

**Keywords:** dynamic spoken language, near-infrared spectroscopy, hyperscanning, two-person neuroscience, neural coupling, acoustical analysis, agreement and disagreement, adaptive models of language

## Abstract

Although the neural systems that underlie spoken language are well-known, how they adapt to evolving social cues during natural conversations remains an unanswered question. In this work we investigate the neural correlates of face-to-face conversations between two individuals using functional near infrared spectroscopy (fNIRS) and acoustical analyses of concurrent audio recordings. Nineteen pairs of healthy adults engaged in live discussions on two controversial topics where their opinions were either in agreement or disagreement. Participants were matched according to their *a priori* opinions on these topics as assessed by questionnaire. Acoustic measures of the recorded speech including the fundamental frequency range, median fundamental frequency, syllable rate, and acoustic energy were elevated during disagreement relative to agreement. Consistent with both the *a priori* opinion ratings and the acoustic findings, neural activity associated with long-range functional networks, rather than the canonical language areas, was also differentiated by the two conditions. Specifically, the frontoparietal system including bilateral dorsolateral prefrontal cortex, left supramarginal gyrus, angular gyrus, and superior temporal gyrus showed increased activity while talking during disagreement. In contrast, talking during agreement was characterized by increased activity in a social and attention network including right supramarginal gyrus, bilateral frontal eye-fields, and left frontopolar regions. Further, these social and visual attention networks were more synchronous across brains during agreement than disagreement. Rather than localized modulation of the canonical language system, these findings are most consistent with a model of distributed and adaptive language-related processes including cross-brain neural coupling that serves dynamic verbal exchanges.

## Introduction

Everyday conversations in a social world are made up of situations in which agreement and disagreement are components of transactions and negotiations communicated by language. While linguists have investigated the behavioral aspects of these interactions (e.g., Pickering and Garrod, 2004; Babel, 2012), and the neural correlates of spoken language exchanges within dyads have been previously described (Jiang et al., 2012, 2017; Hirsch et al., 2018) understanding how neural systems adapt to extended dialectical discussions between partners remains an open and timely research area. The relevance of insight regarding the neurobiology of human dyadic behavior during expressions of congruent and incongruent opinions is highlighted in times of extreme political and social division. In this study a paradigm of face-to-face verbal “debate” is applied to compare neural systems engaged during expressions of agreement or disagreement. As in formal debates each participant was given a limited amount of time, here 90 s in alternating 15 s turns, to “make their case.” A conventional notion of the functional architecture of the brain is based on the assigned functions of isolated regions. This theoretical framework predicts that neural responses to these two conditions would differ by modulating activity in core language-related regions. However, an alternative approach based on a constructionist model (Lindquist and Barrett, 2012) would predict that multiple functional networks in addition to the language system would dynamically adapt to the emerging social situation. Although not necessarily mutually exclusive, these two alternatives encompass a range of the unanswered questions related to dynamic language used in realistic social situations.

Language, visual, and social systems are dynamically intertwined, and are typically investigated and modeled as properties of isolated and functionally specialized systems within single brains. Classical models of speech generation (e.g., Levelt, 1989), and models that provide explicit mapping of language functions onto brain regions (Guenther, 1995, Hickok and Poeppel, 2000), do not directly consider either verbal or visual input from an interlocutor as a potential source of modulation. While these single-brain and single-system approaches have informed prevailing views of how the visual, social, and language systems are organized, it has been suggested that further advances in understanding are likely to emerge from observations within the interactive social settings (e.g., Schilbach, 2010; Hasson et al., 2012; Schilbach et al., 2013). Investigation of neural systems that underlie dynamic social interactions between paired rather than single individuals challenges conventional methods and paradigms (Koster-Hale and Saxe, 2013; Redcay and Schilbach, 2019; Wheatley et al., 2019). Functional near-infrared spectroscopy (fNIRS) is a neuroimaging technique that supports observation of simultaneous objective measures of hemodynamic signals during live, person-to-person, verbal interactions under naturalistic conditions (Jiang et al., 2012; Babiloni and Astolfi, 2014; García and Ibáñez, 2014; Schilbach, 2014; Pinti et al., 2015, 2018, 2020; Hirsch et al., 2018) and is applied in this investigation to address these issues. Technical advances that enable the acquisition of brain signals acquired simultaneously on two people during live and natural interactions have catalyzed research focused on live dyadic behavior (Villringer and Chance, 1997; Ferrari and Quaresima, 2012; Boas et al., 2014). Although the spatial resolution of fNIRS is limited to ~3 cm, tolerance to head movement is sufficient for acquisition of valid hemodynamic signals under natural conditions.

The perception of a dynamic face in experimental isolation is known to incorporate many complex factors that are interpreted in real time (Lachat et al., 2012; Koike et al., 2016, 2019; Chang and Tsao, 2017). Neural activity specific to perception of faces has been observed in the inferior occipital and fusiform gyri, while perception of dynamic eye gaze has been associated with higher processing areas in the superior temporal sulci and temporoparietal junction (TPJ) (Haxby et al., 2000; Hoffman and Haxby, 2000; Pitcher et al., 2011; Sato et al., 2016). The mechanism of information exchange and regulation of circuits that up-regulate communication related to real and dynamic faces in social interaction is not well-understood (e.g., Richardson and Dale, 2005). However, it is widely appreciated that these networks include the right TPJ, fusiform face area, occipital face area, and the posterior superior temporal sulcus (George et al., 2001; Hooker et al., 2003; Mosconi et al., 2005; Pelphrey et al., 2005; Sorger et al., 2007; Saito et al., 2010; Cavallo et al., 2015). Anterior temporal gyrus and prefrontal lobe structures have also been shown to play a role in these interactions including the inferior and medial frontal gyri (Duchaine and Yovel, 2015).

Current understanding of the neural activity that underlies spoken language is primarily based on evidence from single brains performing language tasks other than real interactive speech. Canonical models assume specialized regions that coordinate receptive and productive functions. For example, frontal regions, including left inferior frontal gyrus, pars opercularis, and pars triangularis (Broca's region) are typically associated with speech production. Temporal-parietal regions including left middle and superior temporal, supramarginal, and angular gyri (Wernicke's region) have been associated with speech reception and comprehension of auditory signals. These systems are typically investigated by fMRI and EEG by employment of internal thought processes (covert speech) rather than actual (overt) speach. This limitation is due primarily to the deleterious effects of movement during speech production using functional magnetic resonance, fMRI, although, in some cases, actual speaking has been achieved during scanning (Gracco et al., 2005; Stephens et al., 2010). However, fMRI technology is restricted to one participant at a time and the acquisition of neural activity during speaking as it occurs in actual live interactive dialogue with another person requires an alternative technology. Advancing this direction of research, functional near infrared spectroscopy, fNIRS, has been successfully applied to investigate and live interactive speaking (Jiang et al., 2012; Scholkmann et al., 2013b; Liu et al., 2017; Zhang et al., 2017; Hirsch et al., 2018; Descorbeth et al., 2020), effectively extending neurobiological models of live verbal communication and social interaction.

Spoken language and visual processes are generally associated with high-level cognitive and linguistic functions (Gabrieli et al., 1998; Binder et al., 2000; Price, 2012). Similarly, social systems have also been associated with high-level core functions including theory of mind, empathy, memory, and attention (Carter and Huettel, 2013). However, understanding how these systems interact and mediate the rapid and dynamic exchanges of visual, linguistic, and social information during live verbal interactions represents an emerging area of investigation that is advanced here by comparing neural responses during verbal agreements and disagreements.

A fundamental question in social and interactive neuroscience relates to how dynamic neural systems adapt to various social conditions. It has been proposed that in a natural dialogue emergent perceptual experiences of dyads are shared in order to achieve a common level of understanding (Garnier et al., 2013). How does this occur? How do the canonical neural systems for language, face processing, and social systems adapt when challenged by agreement or disagreement with another person? Does agreement during a dialogue or alternatively disagreement modulate systems associated with theory of mind (Saxe and Kanwisher, 2003; Saxe and Powell, 2006)?

To address these questions, we compare patterns of neural activity from extended verbal dialogues that contrast pre-assessed mutual agreement or disagreement. In such an interaction “the dyad” functions as a social unit where both individuals are “linked” together by the exchange of spoken information in the foreground and a simultaneous stream of on-going social cues such as face and acoustic processing occurring in the background. During normal conversation these “back-channel” cues are used to signal understanding, affect, and willingness to cede or accept the speaking role (Schegloff et al., 1974). Their effect is such that, although subject to cultural variation, human speakers tend to take turns in conversation so that overlap of speech is avoided and inter-speaker silence is minimized. For example, across the ten typologically different languages examined by Stivers et al. (2009) the mean inter-speaker gap was ~200 ms. This short latency between turns suggests that talking and listening functions occur nearly simultaneously during typical conversation where listeners necessarily formulate their response before the incoming speech has been completed. Thus, talking and listening, as it occurs in a natural dialogue, can be modeled as a single two-person (dyadic) function where processes for “sending” and “receiving” implicit and explicit information occur simultaneously and reciprocally.

Converging patterns of coordinated behavior are frequently observed during interpersonal interactions such as walking in step (Zivotofsky and Hausdorff, 2007) or synchronized applause (Néda et al., 2000). One model for dynamic and cooperative behaviors during dialogue, a very specific example of coordinated behavior, proposes a counter-phased pattern of intention to speak that is driven by a common syllable rate between dyads such that oscillating processes within the brains of speaker and listener are mutually entrained (Wilson and Wilson, 2005). Such a framework predicts neural entrainment between the two interacting brains. Empirical support for this idea of neural coupling by mutual entrainment during a language task has been provided by a functional MRI study (Stephens et al., 2010) in which the brain activity of a speaker was first measured while they told an unrehearsed life story; next the brain activity of a listener was measured while they heard the recorded audio of the story; and finally the listener's comprehension of the story was assessed using a detailed questionnaire. Using inter-subject correlation analysis Stephens et al. found that the speaker's and listener's brains exhibited joint, temporally aligned patterns of neural coupling that were correlated with the extent of comprehension (e.g., such patterns were significantly diminished when the story was in a language unknown to the listener). These findings suggest that neural coupling between the speaker and listener represent not only oscillatory patterns indicating speaking and listening turn taking behaviors, but also the transfer of mutual information (Dumas et al., 2010; Hasson and Frith, 2016).

The neural substrates of synchronized neural activity of language systems have been investigated by separating recitation and listening to the same story (Stephens et al., 2010; Liu et al., 2017). A hierarchy of activation for the compound epoch (a dyadic situation with both talking and listening functions occurring simultaneously) was associated with multiple levels of perceptual and cognitive processes, and were assumed to operate in parallel with the multiple timescales of representation (Hasson et al., 2012). These observations contributed to the proposal that live communication between dyads includes neural coupling of rapidly exchanged signals that also convey information (Gregory and Hoyt, 1982; Hasson and Frith, 2016). Further empirical support for this theoretical framework was found in a subsequent hyperscanning investigation of simultaneous talking and listening during a natural dialog paradigm (Hirsch et al., 2018). Increased neural coupling was observed between the superior temporal gyrus (BA 42) and the adjacent subcentral area (BA 43) during talking and listening with interaction relative to the monolog (non-interactive) condition. Together this theoretical framework and these empirical findings support a biological underpinning for dynamic interactive behavior that is evidenced by neural coupling during language tasks.

The focus on neural coupling between communicating individuals during natural face-to-face dialogue highlights the dyad as a dynamic functional unit within this theoretical framework. For example, in this case, the dyad takes on properties that are not necessarily true of either individual alone. These properties may include shared and reciprocal representations of dynamic information transfers modified by both implicit and explicit information. This approach marks a departure from a conventional single brain investigation designed to investigate modular and specialized processes such as encoding faces and linguistic communications. Here we focus on the neural underpinnings of dyadic interactions engaged in a live and face-to-face verbal ‘debate’ where agreement and disagreement of personally held opinions are expressed. The neural effects are compared under the hypothesis that these multimodal behaviors reveal high level integrated processes of neural adaptations and organization.

## Materials and Methods

### Participants

Thirty-eight healthy adults (19 pairs, 23.7 ± 4.43 years of age, 19 female, 37 right-handed (Oldfield, 1971) participated in the experiment and all were included in the analysis. Ten dyads were mixed genders. Invitations to participate were distributed throughout the Yale University campus and surrounding areas. Participants were informed that the experiment was aimed at understanding the neural underpinnings of interpersonal communication, and provided informed consent prior to the investigation in accordance with the Yale University Human Investigation guidelines (HIC protocol no.1501015178).

Pre-existing opinions of participants were assessed by an online questionnaire using SurveyMonkey. Each participant rated their views on controversial topics involving politics, ethics, philosophy, health, and environment. The questionnaire included 30 statements such as “same-sex marriage is a civil right,” “marijuana should be legalized,” “the death penalty should be banned,” and “video games are a waste of time.” A 5-point scale indicated degree of agreement or disagreement: 1 (Strongly Disagree); 2 (Disagree); 3 (Neither Agree nor Disagree); 4 (Agree); and 5 (Strongly Agree). Responders also indicated whether they were willing to discuss the topic or not. Based on these responses, pairs of participants (dyads) were selected based on the criteria that each pair agreed or strongly agreed on two topics and also disagreed or strongly disagreed on two others. The dyads were selected based on the differences between scores for each topic such that high differences (3 and 4) indicated two levels of disagreement and low differences (1 and 2) indicated two levels of agreement. Specifically, in this sample, 60% of the agreements were the higher level 1 (Strongly Agree) and 40% were the lesser level 2 (Agree); whereas 67% of the disagreements were the lesser level 3 (Disagree); and 14% were the higher level 4 (Strongly Disagree). Thus, overall, the ratings suggest a slight bias toward agreement relative to disagreement. This was a “within subjects” design because each dyad agreed on two topics and disagreed on two others. The topic assignments for the participants were taken from the Survey Monkey statements and were thus aligned with their stated views. Confirmation that the conversations between partners were on the assigned topics with the expected turn-taking was obtained from the voice recordings that were transcribed and reviewed by two independent investigators. The statements for discussion were unique for each dyad as they were determined by the *a priori* individual ratings. In all cases the participants were strangers or casually acquainted as classmates prior to the experiment. All dyads were unique and there were no cases of individuals who participated in more than one experiment. Participants were informed that the experiment was an investigation of verbal communication.

### Experimental Procedures

#### Task

Dyads were assigned four topics for discussion according to their SurveyMonkey responses; however, the topic for discussion was not known to the participants until immediately before the start of each run. The orders of the speakers and topics were randomized. Participant opinions were not known to participants or investigators at the time of the experiment as the orders and topics had been coded by a numbering system during the dyad-planning stage. Thus, any and all social and opinion signifiers were detected by natural and undirected processes. The paradigm was intended to simulate a spontaneous social situation similar to that where strangers might happen to begin a conversation while sitting next to each other on a bus, and discover that they were either in agreement or not with respect to a specific topic. However, to ensure equal time for both participants and to support the block design necessary for fNIRS recording, turn-taking was imposed through a timing mechanism similar to that used in formal debates, in which each speaker alternates speaking turns on a pre-set schedule.

All dyads discussed/debated two topics on which they naturally agreed and two topics on which they naturally disagreed in the same setting. Thus, both conditions of agreement and disagreement were similarly influenced by the same “subjective variables” such as judgments regarding appearance, style, gender, age, and ethnicity, etc. We assume that this “within-dyad” control technique, averaged over the group of participants, was sufficient to assure that observed effects are due to the manipulations of agreement and disagreement rather than arbitrary effects of social pairing.

#### Experimental Paradigm

**Figure 1:**
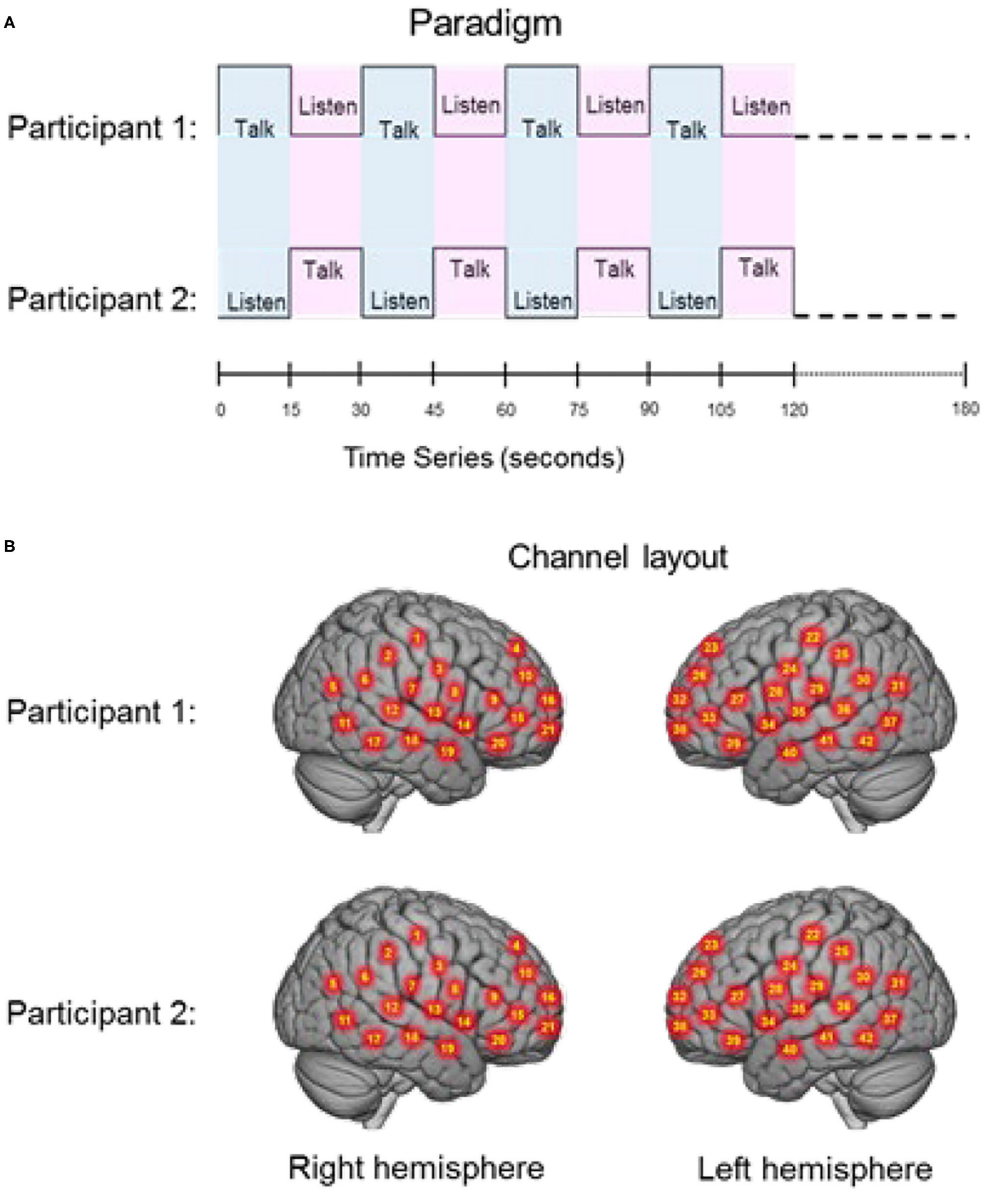
**(A)** Experimental paradigm. Participants alternated between talking and listening to each other every 15 s of each 180 s run (see Hirsch et al., 2018); **(B)** Right and left hemispheres of rendered brains illustrate average locations (red circles) for the 42 channels per participant identified by number. Montreal Neurological Institute (MNI) coordinates were determined by digitizing the locations of the optodes in relation to the 10–20 system based on conventional landmarks. See Supplementary Table 1 for group median coordinates, anatomical regions, and atlas-based probabilities for each channel.

The experimental paradigm was similar to previously reported two-person verbal interaction paradigms (Hirsch et al., 2018; Descorbeth et al., 2020). Participants were positioned ~140 cm across a table from each other with a full view of their partner's face. Each wore an array of “optodes” (small detectors and emitters for the acquisition of hemodynamic signals) providing extended head coverage over both hemispheres of both participants and a head-mounted camera and microphone from which audio was sampled at 44.1 kHz. The experiment consisted of a total of four 3 min runs. The time series is shown in Figure 1A. “Speaker” and “Listener” roles switched every 15 s, such that each participant had the speaking role for six of the 12 total turns per run. The change in speaker/listener roles was indicated by a tone and also by small green and red lights displayed in front of the participants indicating turns for talking or listening, respectively. The light and tones used to signal turns were controlled by PsychoPy and the triggers for all synchronization were controlled by a custom built TTL based triggering device. A separate conversation topic was assigned prior to each run, and the order of the topics was randomized across dyads. The first speaker was assigned by the investigator, and subsequent speaker order was alternated between runs. Although the transition between speaker and listener was established by this paradigm, speakers were permitted to finish sentences at the “turn-taking boundaries” in order to maintain a sense of a natural conversation. A total of 74 “debates” were recorded (37 agree and 37 disagree). The audio was not recorded for one pair. Thus, the full data set consists of 19 pairs and the audio data set thus consists of 18 pairs.

### Signal Acquisition and Processing

#### Functional NIRS Signals

Signal acquisition methods have been described previously (Hirsch et al., 2018; Descorbeth et al., 2020) and are included here to provide a self-contained report. Hemodynamic signals were acquired using a 64-fiber (84-channel) continuous-wave fNIRS system (LABNIRS, Shimadzu Corp., Kyoto, Japan) setup for hyperscanning of two participants. Figure 1B illustrates the spatial distribution of 42 channels over both hemispheres of each participant. Temporal resolution for signal acquisition was 27 ms. In the LABNIRS system, three wavelengths of light (780, 805, and 830 nm) are delivered by each emitter, and each detector measures the absorbance for each of these wavelengths. These wavelengths were selected by the manufacturer for differential absorbance properties related to the oxygen content of blood. The absorption for each wavelength is converted to corresponding concentration changes for deoxyhemoglobin, oxyhemoglobin, and for the total combined deoxyhemoglobin and oxyhemoglobin. The conversion of absorbance measures to concentration have been described previously (Matcher et al., 1995).

##### Optode Localization

The anatomical locations of optodes were determined for each participant in relation to standard head landmarks including inion; nasion; top center (Cz); and left and right tragi using a Patriot 3D Digitizer (Polhemus, Colchester, VT), and linear transform techniques as previously described (Okamoto and Dan, 2005; Eggebrecht et al., 2012; Ferradal et al., 2014; Hirsch et al., 2018; Descorbeth et al., 2020). The Montreal Neurological Institute, MNI, coordinates for the channels were obtained using the NIRS-SPM software (Ye et al., 2009) with MATLAB (Mathworks, Natick, MA), and corresponding anatomical locations of each channel were determined and are shown in Supplementary Table 1.

#### Signal Processing and Global Component Removal

Pre-processing. Baseline drift was removed using wavelet detrending (NIRS-SPM). Any channel without a signal was identified automatically by the root mean square of the raw data when the signal magnitude was more than 10 times the average signal.

Global component removal. Global systemic effects (e.g., blood pressure, respiration, and blood flow variation) have previously been shown to alter relative blood hemoglobin concentrations (Boas et al., 2004). These effects are represented in fNIRS signals and introduce the possible confound of acquiring hemodynamic responses that are not due to neurovascular coupling (Tachtsidis and Scholkmann, 2016). These non-neural global components were removed using a principle components analysis (PCA) spatial filter (Zhang et al., 2016, 2017) prior to general linear model (GLM) analysis. This technique exploits advantages of the distributed optode coverage in order to distinguish signals that originate from local sources (assumed to be due to the neural events) by removing global signal components. assumed to originate from systemic cardiovascular events) (Zhang et al., 2016; Hirsch et al., 2018; Descorbeth et al., 2020; Noah et al., 2020).

##### Hemodynamic Signals

Both OxyHb and deOxyHb signals acquired by fNIRS provide a hemodynamic proxy of neural activity. However, the OxyHb signal has been shown to be more sensitive to global components than the deOxyHb signal due to systemic effects directly related to non-neural factors such as blood pressure, respiration, and blood flow (Kirilina et al., 2012; Tachtsidis and Scholkmann, 2016; Zhang et al., 2016). The deOxyHb signal, on the other hand, is theoretically more closely related to the paramagnetic effects of deOxyHb acquired by fMRI (Ogawa et al., 1990) and is characterized by lower signal-to-noise than the OxyHb signal (Strangman et al., 2002). Further, recent reports confirm that speaking during fNIRS studies produces changes in arterial CO_2_ that alter the OxyHb signal to a greater extent than the deOxyHb signal (Scholkmann et al., 2013b,c; Hirsch et al., 2018; Descorbeth et al., 2020).

The choice of reporting the deOxyHb signal for this study was based on the above prior findings and also by a “localizer method” employed to document expected locations of activity. In this study all conditions with talking and listening were combined to localize the canonical language areas associated with speech production (Broca's Area) and language reception (Wernicke's Area) on the left hemisphere. Both signals, deoxyhemoglobin, deOxyHb, and Oxyhemoglobin, OxyHb, with and without spatial filtering, a processing technique employed to remove non-neural signal components as described above (Zhang et al., 2016) were compared and evaluated for the presence of the known “localizers.” These localizers were observed for the deOxyHb signal with the filtered preprocessing, and is the signal reported in this investigation. Similar approaches have been employed in other investigations of natural verbal exchanges between dyads (Zhang et al., 2017; Hirsch et al., 2018; Descorbeth et al., 2020).

### Statistical Analysis of fNIRS Data

#### General Linear Model (GLM)

Contrast effects were based on comparisons of talking vs. listening and determined by a voxel-wise approach as conventionally applied to fMRI and adapted for fNIRS (see Hirsch et al., 2018; Descorbeth et al., 2020) for further details of this approach). Reported findings were corrected by the False Discovery Rate (FDR) method at a threshold of *p* < 0.05. The 42-channel fNIRS datasets per subject were reshaped into 3-D volume images for the first-level general linear model (GLM) analysis using SPM8 where the beta values were normalized to standard MNI space using linear interpolation. All included voxels were within 1.8 cm from the brain surface. The computational mask consisted of 3,753 2 × 2 × 2 mm voxels that “tiled” the shell region covered by the 42 channels. In accordance with this technique the anatomical variation across subjects was used to generate the distributed response maps. Results are presented on a normalized brain using images rendered on a standardized MNI template and with techniques validated for reliability using fNIRS (Dravida et al., 2017). Anatomical locations of peak voxel activity were identified using NIRS-SPM (Ye et al., 2009). Talking [talking > listening] and listening [listening > talking] functions are compared for agree and disagree conditions.

This analysis includes a co-regressor based on Area under the Speech Envelope, ASE, as a proxy for acoustic energy which is convolved with the canonical hemodynamic response function, hrf. Evaluated over a short (100 ms) window, this regressor served to remove short-term (syllable-level) effects related to the (spontaneous) choice of words by each speaker that might otherwise interfere with the turn-length neural patterns of interest associated with agreement or disagreement. The dyadic nature of this paradigm establishes a situation where talking and listening occur simultaneously. Although each partner is alternately engaged in one role or the other, the sending and receiving of visual and verbal social information can be characterized as simultaneously driving reciprocal perceptions that prompt a cascade of interrelated actions and reactions. This reverberating “closed loop” informs the comparison of the neural coupling that occurs during these two interrelated functions.

#### Cross-Brain Neural Coupling

Cross-brain synchrony (coherence) was evaluated for both the agree condition and the disagree condition using wavelet analysis (Torrence and Compo, 1998; Cui et al., 2011) from the MATLAB 2018A Wavelet Toolbox. The wavelet kernel was a complex Gaussian (Mexican hat-shaped kernel) provided by MATLAB. The range of frequencies was 0.4–0.025 Hz consistent with the timing of the hemodynamic response function (Zhang et al., 2020). Wavelet analysis with these parameters requires a 10 s cycle and three cycles are needed for a wavelet. Therefore, our highest resolution is 30 s and that includes both the talking (15 s) and listening (15 s) turns. While amplitude and phase are both components of coherence analysis, we consider only the amplitude in this analysis. Cross-brain coherence between dyads was measured between pairs of brain regions. Individual channels for each participant were grouped into 15 anatomical regions based on shared anatomy. For most participants there were two or three channels per region. Grouping was achieved by identification of 15 bilateral ROIs from the acquired channels including: (1) angular gyrus (BA39); (2) dorsolateral prefrontal cortex (BA9); (3) dorsolateral prefrontal cortex (BA46); (4) pars triangularis, BA45; (5) supramarginal gyrus (BA40); (6) occipitotemporal cortex (BA37); (7) middle temporal gyrus (BA21); (8) superior temporal gyrus (BA22); (9) somatosensory cortex (BA1, 2, and 3); (10) somatosensory association cortex (BA7); (11) pre-motor and supplementary motor cortex (BA6); (12) subcentral area (BA43); (13) inferior frontal gyrus (BA47); and (14) visual cortex (Area V3, BA19) (15) frontal eye fields (BA8). Methodological details and validation of this technique have been previously described (Hirsch et al., 2017, 2018; Descorbeth et al., 2020; Zhang et al., 2020).

Previous investigations by our group observed that sub-regions within the temporal parietal junction, TPJ (known as a complex for social processing) including the SMG, AG, and STG, are coherent during face to face interactions (Noah et al., 2020). We hypothesized that these specialized mechanisms for rapid exchanges of information such as facial cues would vary during this experiment due to factors such as voice and the face-to-face variations expected during dialogues with agreement and disagreement. This hypothesis defined the entirety of the TPJ as a region of interest (as we do not yet have a specific hypothesis about the respective roles of the anatomical subcomponents). Signals acquired from these predefined anatomical regions were decomposed into a range of temporal frequencies that were correlated across two brains for each dyad. This technique effectively removes the task regressor employed with General Linear Model, GLM, approaches as is conventional for Psychophysiological Interaction (PPI) analysis (Friston, 1994; Friston et al., 2003; McLaren et al., 2012). Here, we apply this “residual signal” to investigate effects other than the main task-induced effect related to cross-brain coherence. In this case, cross-brain coherence of signal components (wavelets) is thought to provide an indication of dynamic neural coupling processes rather than task-specific processes which are synchronized by virtue of the alternating experimental conditions. The experimental period was 30 s (15 s talk and 15 s listen and both functions occurred simultaneously during each epoch). Statistical *t*-tests were applied for comparison of coherence between the agree and disagree conditions on three successive bins of periods. The risk of a false positive finding due to multiple *t*-tests between conditions is minimized by the limited wavelength range within the hemodynamic response function that we consider, 10–20 s (i.e., three bins), and the decision rule that significant effects were based on at least two consecutive results. Previous findings indicate that coherence of fNIRS signals is expected to maximally manifest in the wavelength range of 10–20 s (Zhang et al., 2020). Coherence during agreement was compared to disagreement conditions. This analysis was also applied to shuffled dyads (random pairs) as a control for non-social effects. If the effects are due to real social exchanges of salient cues, then these effects can be expected to disappear when the partners are mixed (shuffled). We present the data for naturally paired dyads and for shuffled pairs to confirm that the measured coherence can be attributed to real effects of the social interaction rather than common experience.

### Behavior Measures: Word-Based Feature Analysis and Acoustical Measures

Audio signals were obtained from recordings of each speaker sampled at 44.1 kHz. Alignment with fNIRS signals was established using an audio tone indicating the end of the debate and the known (10 + 180s) fNIRS sampling duration (for eight debates this was unavailable, and was determined using the final turn swap plus 15 s instead). Concurrent speech was limited to occasional backchannel interjections by the listener (e.g., affirmative “uh-huh”). Microphones were omnidirectional components of head-mounted cameras, and thus insufficiently directional to exclude these; however, in general, participants followed directions to speak only during their respective turns. In instances where speakers started their turn early or late with respect to the transition signal, the acoustic labeling was based on the actual rather than the absolute timing of the exchange. Analyses of voice recordings provided two behavioral measures of dyadic agreement and disagreement, word-based feature extraction and acoustical analysis.

#### Word-Based Feature Analysis

Language components that have previously been associated with agreement and disagreement (Hillard et al., 2003) were identified for each recording by two independent raters. Agreement cues included words like “yes,” “I agree,” “absolutely,” “that's true,” “sounds great,” etc., and disagreement language cues included words like “no,” “I disagree,” “you are wrong,” “but,” “that does not answer the question,” etc. Any backchannel cues by the listener were not included. Similar word-based feature extraction methods have been validated for automated techniques to recognize agreement and disagreement utterances in boardroom meetings (Hillard et al., 2003), in conversational speech (Galley et al., 2004), and in multi-party conversation (Germesin and Wilson, 2009). This classifier system was adapted to distinguish the general nature of the conversations in this experiment as either agreement or disagreement. Combined summaries of transcribed narratives included counts of positive and negative “key words.”

#### Acoustical Analysis

F0 contours for each turn were computed using Praat (Boersma, 1993), with values for male speakers doubled to facilitate comparison with female speakers. A syllable count for each turn was obtained using an implementation of the RMS-based algorithm of Mermelstein (1975). Acoustic energy was obtained by integrating the speech envelope determined by the smoothed (50 ms window) root-mean-square (RMS) signal derived from the decimated (×100) turn audio. The following behavioral measures were computed for each speaker turn:

- Syllable Rate (SYLR): turn syllable count normalized by turn duration (syllables/sec)
- Median Fundamental Frequency (MF0): median F0 over turn (Hz)
- Fundamental Frequency Range (RF0): F0 range over the turn in Hz, after discarding values ±1.5 times the interquartile range
- Area under Speech Envelope (ASE): area under speech envelope normalized by turn duration (acoustic energy); also computed at a 10 Hz rate for use as a GLM regressor.

Measures were computed in Matlab and analyzed in R Core Team (2018) using the *lme4* (Bates et al., 2015) and *lmerTest* (Kuznetsova et al., 2017) packages. To enhance the within-speaker contrast between debate conditions measures were mean-centered and divided by standard deviation (z-scored) over all values in all debates for each speaker. Linear mixed-effects models (LMM) were used to predict each measure from fixed effects of BINS (grouping first three and last three turns) and AGREE (Y/N) as fixed effects and including random slopes and intercepts by speaker (binning over turns was used to observe changes in response over the course of the debate). The interaction term did not improve model fits and was not included. A one-sided paired *t*-test (H_1_: agreeN > agreeY) paired measures from corresponding turns from the same speaker in agree vs. disagree debates. In addition, a mixed-effects logistic regression model was used to predict debate agreement from the four measures simultaneously as continuous covariates, with random intercepts by speaker.

## Results

### Signal Validation and Regions of Interest (ROI)

**Figure 2:**
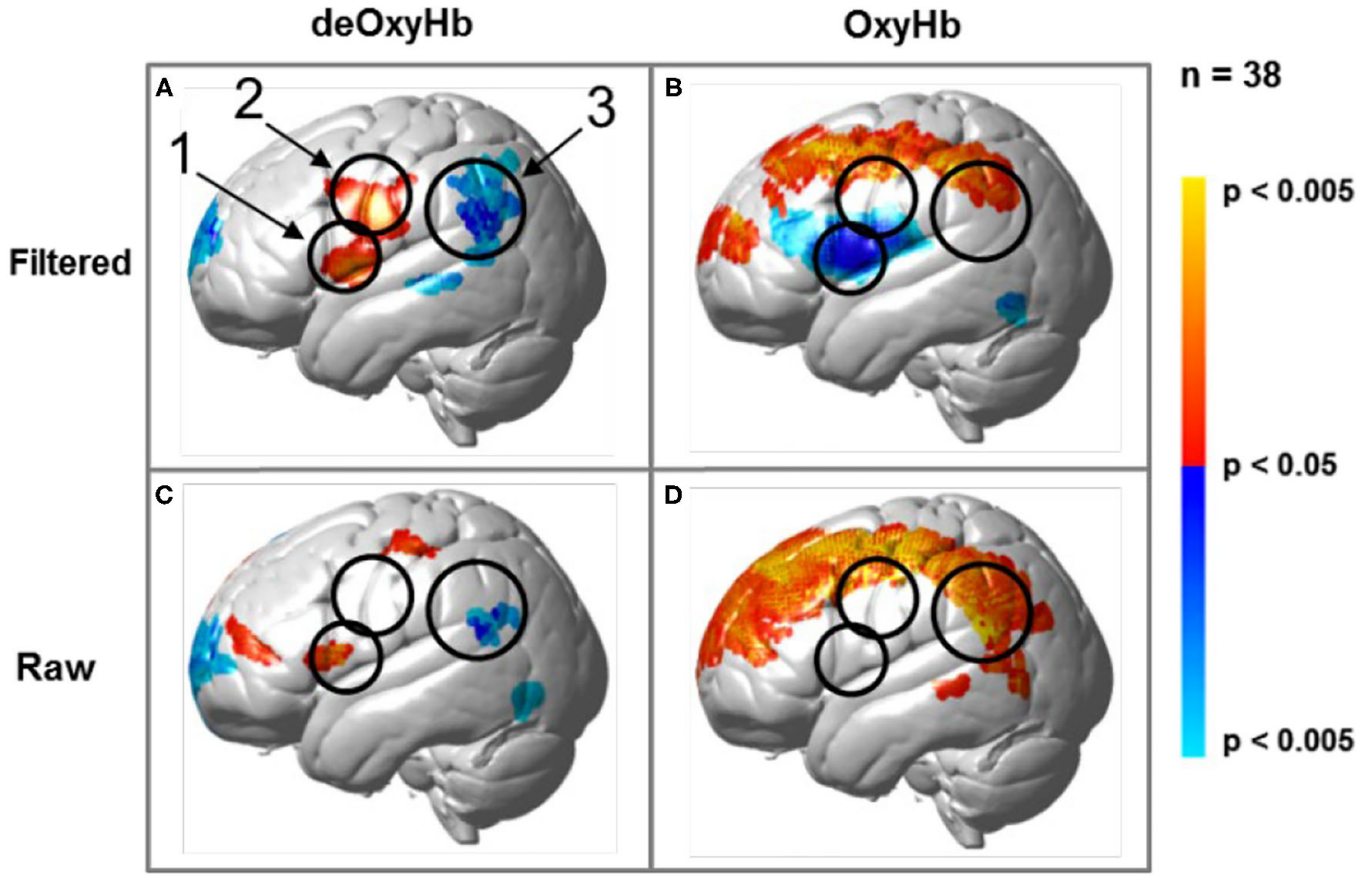
Signal selection was based on empirical comparison of voxel-wise contrasts from deOxyHb (left column) and OxyHb (right column) signals with global mean removal (Filtered, top row) and without global mean removal (Raw, bottom row). Red/yellow indicates [talking > listening], and blue/cyan indicates [listening > talking] with levels of significance indicated by the color bar on the right. Images include left hemisphere sagittal views. The black circles represent the canonical language regions, Broca's Area (anterior), the articulatory sensory-motor system (central), and Wernicke's Area (posterior). The specific Regions of Interest (ROI) are indicated as 1, 2, and 3, respectively. See Table 1. These regions are observed for the deOxyHb signals following global mean removal (Zhang et al., 2016, 2017) shown in panel **(A)**, but not for the other signal processing approaches, as illustrated in panels **(B–D)** by the absence of expected localized activity. This empirical approach supports the decision to use the spatially filtered deOxyHb signal for this study and is consistent with similar findings of previous talking and listening experiments (Hirsch et al., 2018).

Regions of brain expected to be associated with talking are left hemisphere inferior frontal gyrus (pars opercularis) and articulatory systems including motor and somatosensory cortex; and regions expected to be associated with listening are middle and superior temporal gyrus, angular gyrus, and supramarginal gyrus, Broca's and Wernicke's Areas, respectively. Validation of these canonical regions using similar fNIRS two-person dialogue techniques has been previously reported (Hirsch et al., 2018). Comparisons of these fiducial markers were made for both OxyHb and deOxyHb signals for unprocessed (Raw) data and with the global-mean removed (Filtered) data, Figure 2. Circled clusters in the upper left panel document observed activity in left hemisphere canonical language production, speaking and articulation (red), and reception, listening (blue) regions. These regions are not seen for the OxyHb signals or raw data shown in the other panels consistent with the established practice of utilizing the deOxyHb fNIRS signals following the spatial filtering technique, when functional tasks include live talking. The anatomical descriptions of the regions circled in panel A for talking functions [Talk > Listen] and listening functions [Listen > Talk] are presented in Table 1. These regions were consistent with expectations of canonical models of human language systems (Gabrieli et al., 1998; Binder et al., 2000; Price, 2012; Hagoort, 2014; Poeppel, 2014). Similar comparisons have led to the use of deOxyHb signals in prior fNIRS investigations of overt speaking (Zhang et al., 2017; Hirsch et al., 2018; Descorbeth et al., 2020); eye-to-eye contact (Hirsch et al., 2017); competitive games (Piva et al., 2017); dyadic communication via interactive drumming (Rojiani et al., 2018); and joint attention (Dravida et al., 2020).

**Table 1:** GLM analysis: group median coordinates, anatomical regions, and atlas-based probabilities [Talking > Listening].

**Contrast**	**Threshold**	**Peak voxels**						**Anatomical regions**	**BA^***c***^**	**Probability**	**n of voxels**
		**MNI coordinates**^*******a*******^			***t***	***p***	**Df^***b***^**				
		**X**	**Y**	**Z**							
[Talk > Listen]	0.04	−62	2	4	2.35	0.012	35	Pre- and supplementary motor cortex	6	0.46	244
								Subcentral area	43	0.26	
ROI 1								Superior temporal gyrus (anterior)	22	0.15	
								Pars opercularis	44	0.14	
		−62	−6	30	2.40	0.011	35	Primary somatosensory cortex	2	0.44	368
ROI 2								Primary somatosensory cortex	3	0.22	
								Primary somatosensory cortex	1	0.22	
								Primary motor cortex	4	0.13	
[Listen > Talk]	0.04	−64	−52	26	−3.41	0.001	35	Supramarginal gyrus	40	0.42	61
								Superior temporal gyrus	22	0.29	
								Angular gyrus	39	0.29	
		−66	−32	0	−2.37	0.012	35	Middle temporal gyrus	21	0.77	36
ROI 3								Superior temporal gyrus	22	0.23	
		−58	−54	42	−2.29	0.014	35	Supramarginal gyrus	40	0.86	18
								Angular gyrus	39	0.14	
		−60	−48	10	−2.23	0.016	35	Superior temporal gyrus	22	0.70	16
								Middle temporal gyrus	21	0.30	

a *Coordinates are based on the MNI system and (−) indicates left hemisphere*.

b *df, degrees of freedom*;

c BA, Brodmann Area identified by the TD ICBM MNI atlas (Maldjian et al., 2003)

*(see Figure 2A)*.

### Behavioral Findings

#### Word-Based Feature Analysis

Relative average frequencies of key agree words were 80% in the debates that occurred during the agreement condition as opposed to 20% for key disagree words, and relative frequencies of key disagree words were 67% in the debates that occurred during the disagreement condition, vs. 33% for key agree words in that condition. Thus, the word feature extraction results, based on key agree and disagree words during the dialogues, were consistent with the experimental conditions.

#### Acoustical Analysis

**Figure 3:**
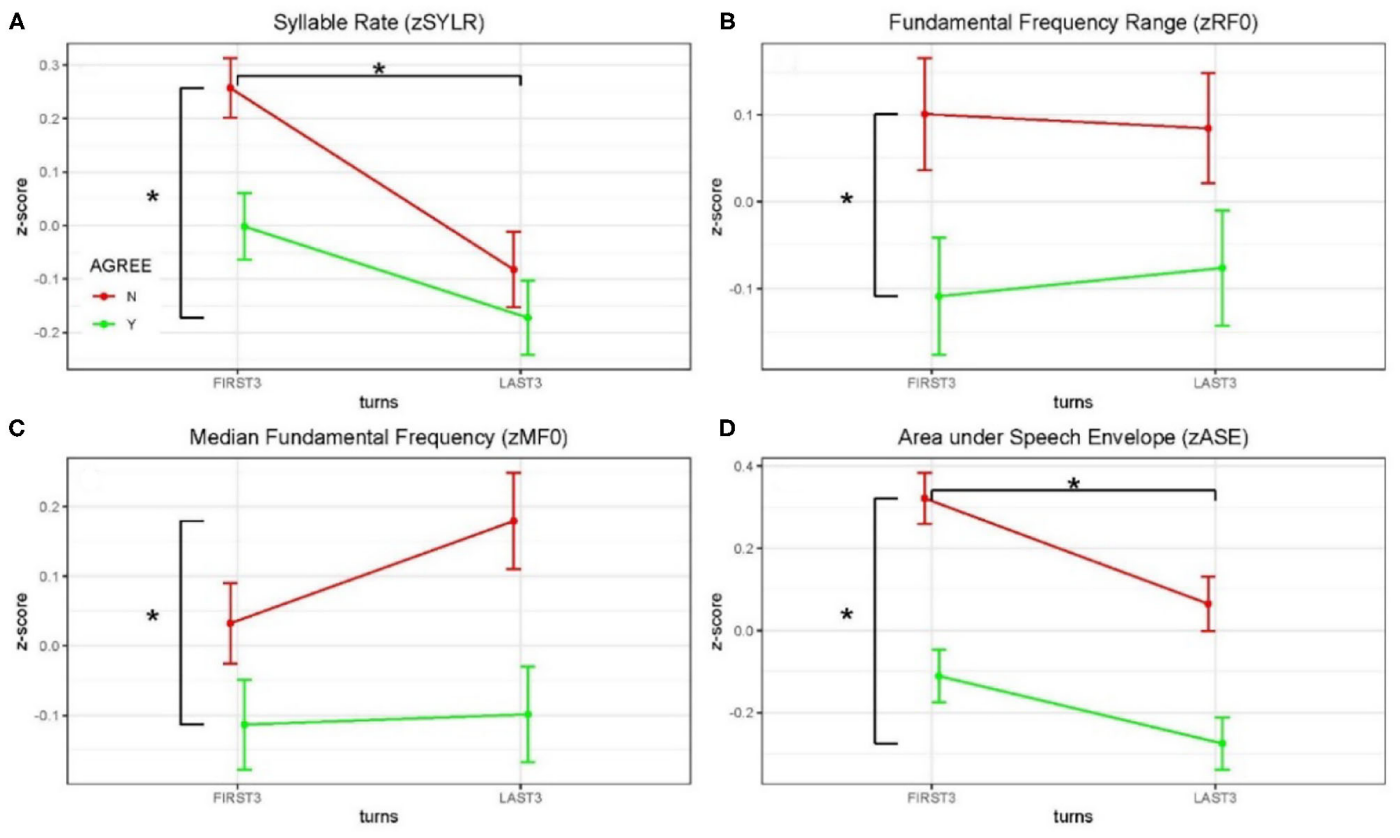
Z-scored audio behavioral measures grouped by agreement (yes or no) and turn (first three turns and last three turns); error bars show standard error of the mean (SEM). Asterisks mark significant comparisons per LMM results. Green represents agree (Yes) and red represents disagree (No). **(A)** Shows syllable rate (syllables/s); **(B)** shows median fundamental frequency (F0) in Hz; **(C)** shows F0 range (Hz); and **(D)** shows area under the speech envelope normalized by turn duration (acoustic energy).

Acoustical data were aggregated across dyads and grouped by debate conditions and first three/last three turns (see Figure 3 and Table 2). Significance of the Linear Mixed-effects Model (LMM) fixed effects was assessed using estimates of the regression coefficients divided by their standard errors (a *t*-test), with degrees of freedom based on the Satterthwaite approximation, and are marked using the *p* < 0.05 ^*^, *p* < 0.01 ^**^ and *p* < 0.001 ^***^ convention, Table 2. A significant difference in syllable rate (zSYLR) was found: speakers spoke more slowly at the end of the debate than during the beginning (t = −3.92^***^); this may reflect flagging inspiration as each discussion wound down. In addition, overall syllable rate was higher for discussions with disagreement (t = 2.68^**^). Overall acoustic energy as represented by the area enclosed by the speech envelope (zASE) showed a similar pattern, with lower energy in the second half of the session (t = −3.28 ^**^), but higher energy overall in debates with disagreement (t = 6.02 ^***^).

**Table 2:** Linear Mixed-effects Model (LMM) results for audio behavioral measures.

	**Coef**.	***t*-value**	***p* est**.	
**zSYLR**: *t*_(443)_ = 2.620, *p =* 0.005**				
(Intercept)	0.040	0.715	0.475	n.s.
AGREE_NO	0.174	2.684	0.007	**
TURNS_LAST3	−0.255	−3.923	0.000	***
**zMF0**: *t*_(443)_ = 3.163, *p =* 0.008***				
(Intercept)	−0.146	−2.583	0.010	**
AGREE_NO	0.211	3.237	0.001	**
TURNS_LAST3	0.081	1.237	0.216	n.s.
**zRF0**: *t*_(443)_ = 2.778, *p =* 0.003**				
(Intercept)	−0.097	−1.705	0.088	∙
AGREE_NO	0.185	2.829	0.005	**
TURNS_LAST3	0.008	0.124	0.901	n.s.
**zASE**: *t*_(443)_ = 5.911, *p =* 0.000***				
(Intercept)	−0.088	−1.583	0.114	n.s.
AGREE_NO	0.386	6.022	0.000	***
TURNS_LAST3	−0.210	−3.281	0.001	**
**Logistic regression**	**Coef**.	***z***–**value**	***p*** **est**.	
(Intercept)	0.001	0.017	0.986	n.s.
zSYLR	−0.099	−1.090	0.276	n.s.
zMF0	0.068	0.850	0.395	n.s.
zRF0	0.184	2.354	0.019	*
zASE	0.476	4.972	0.000	***

Significance was assessed using estimates of the regression coefficients (Coef.) divided by their standard errors (a t-test) with degrees of freedom based on the Satterthwaite approximation. Asterisks indicate significant comparisons using the p ≤ 0.05*, p ≤ 0.01** and p ≤ 0.001*** convention. Non-significant values are marked as n.s. See Figure 3.

*SYLR, syllable rate (syllables/sec); AGREE_NO, debates with topics on which speakers disagreed; TURNS_LAST3, last three turns of each debate; MF0, median fundamental frequency; RF0, fundamental frequency range; ASE, area under the speech envelope (acoustic energy)*.

The median fundamental frequency measure (zMF0) showed significantly higher values overall when speakers disagreed (t = 3.24^**^). In addition, F0 range (zRF0) was significantly wider overall in debates when speakers disagreed (t = 2.83^**^; no effect of session half). The one-sided (agreeN > agreeY) paired *t*-tests comparing measures from corresponding turns in agree vs. disagree debates support this pattern of results (DOF = 403): zSYLR (t = 2.62^**^), zMF0 (t = 3.16^***^), zRF0 (t = 2.78^**^), zASE (t = 5.91^***^). Finally, the results of logistic regression predicting agree/disagree from all four measures showed significant contributions from zRF0 (z = 2.35^*^) and zASE (z = 4.97^***^), with the odds ratio for zASE (acoustic energy) indicating that a one-unit increase predicts a 1.6 greater likelihood of disagreement.

Higher overall F0 and greater F0 range are consistent with enhanced emphasis (e.g., Williams and Stevens, 1972), and are presumably associated with arguing in conflict. In summary, acoustic analysis of the debate audio shows systematic differences in speaker F0 and acoustic energy measures partially contingent on turn (first/last half of the debate), and strongly contingent on debate concordance (agreement/disagreement). These behavioral results are consistent with enhanced speaker emphasis and engagement for the disagreement relative to the agreement conditions, and we take this as behavioral evidence in addition to the topic ratings as consistent with the conditions of agreement and disagreement.

### Neuroimaging Findings

**Figure 4:**
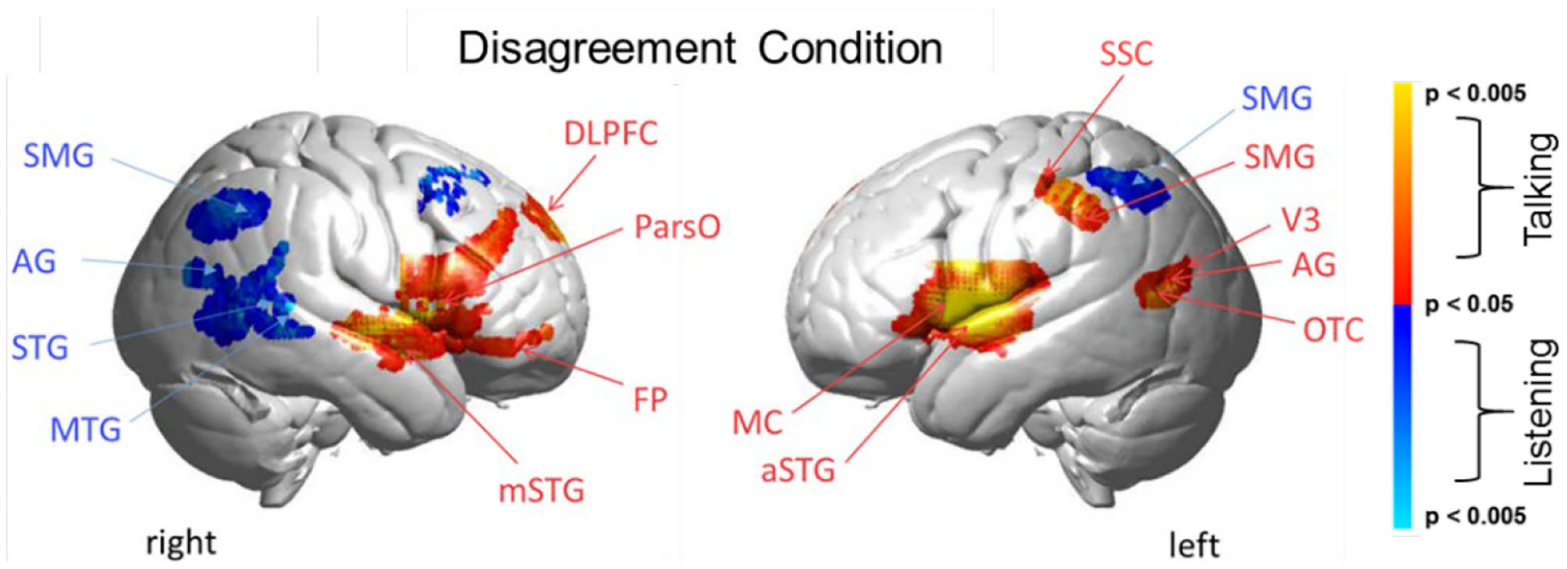
Disagreement condition. Brain activity clusters represent functions of talking [talking > listening] (red) and listening [listening > talking] (blue). Talking (relative to listening) during disagreement with a partner engages the canonical language system including components of left hemisphere Wernicke's Area (AG, angular gyrus; SMG, supramarginal gyrus); Broca's Area (aSTG, anterior superior temporal gyrus; IFG, inferior Frontal gyrus); and components associated with the MC, motor cortex. These frontal systems are also observed bilaterally and include mSTG, middle superior temporal gyrus; ParsO, pars opercularis; DLPFC, dorsolateral prefrontal cortex; FP, frontopolar cortex. Listening during disagreement (blue) engages regions in the right TPJ, temporoparietal junction; AG, including angular gyrus; STG, superior temporal gyrus; MTG, middle temporal gyrus; SMG, bilateral supramarginal gyrus. See Table 3.

**Figure 5:**
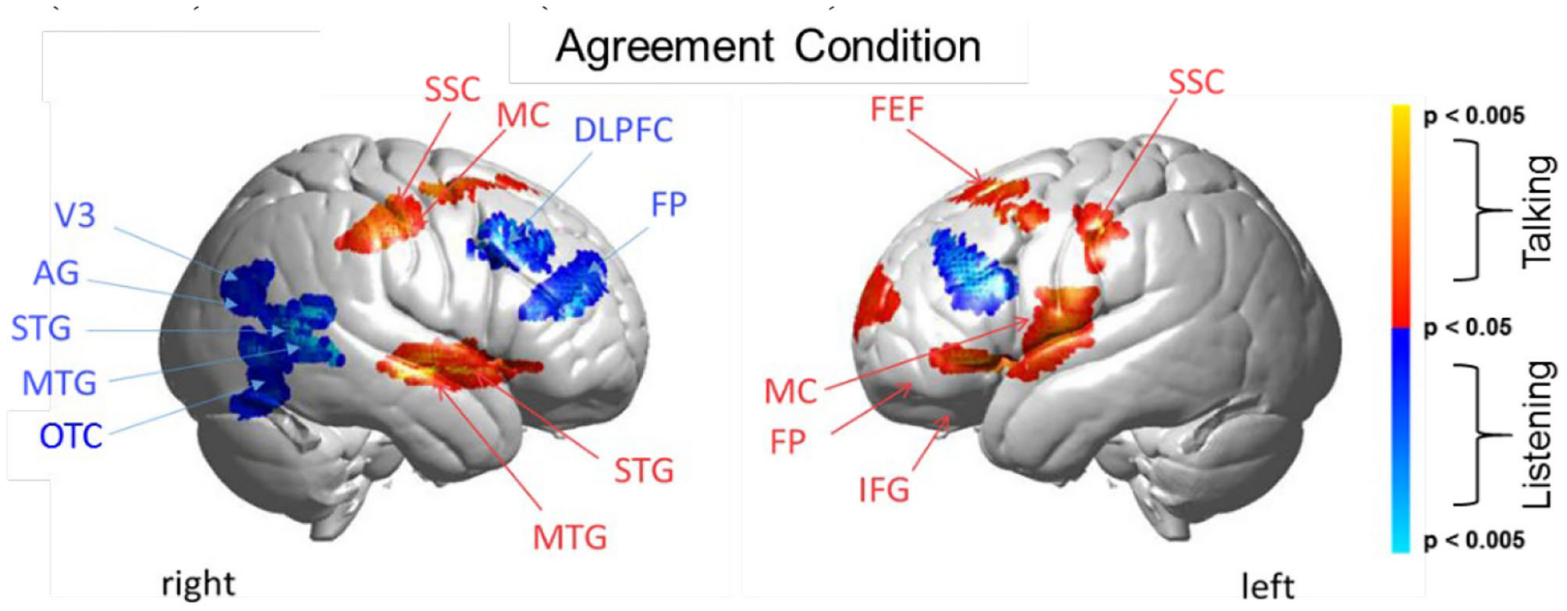
Agreement condition. Brain activity clusters represent functions of talking [talking > listening] (red) and listening [listening > talking] (blue). Talking (relative to listening) during agreement with a partner engages the left frontal language system including components of Broca's Area (anterior STG, superior temporal gyrus; IFG, inferior frontal gyrus; MC, motor cortex; FP, frontopolar cortex). Frontal systems engaged while talking are also observed bilaterally and include FEF, frontal eye fields; SSC, somatosensory cortex; MC, motor cortex. Listening during agreement (blue) engages regions in the right TPJ, temporoparietal junction; including AG, angular gyrus; STG, superior temporal gyrus; MTG, middle temporal gyrus; as well as visual cortex (V3) and face processing regions in the OTC, occipitotemporal cortex. Bilateral DLPFC, dorsolateral prefrontal cortex; FP, frontopolar cortex are also observed during listening when the partners are in agreement. See Table 4.

Main effect contrast comparisons based on the General Linear Model (GLM) are shown for [talking > listening] and illustrated as red clusters, and for the [listening > talking] condition are shown as blue clusters for the disagreement condition (Figure 4). The same convention applies for the agreement condition (Figure 5). The speech energy measure, Area under the Speech Envelope (ASE), was applied as a real-time co-regressor with the intended over-all computational effect of emphasizing the main effect of opinion-based processes and of minimizing possible artifacts due to short-term (syllable-level) effects related to the spontaneous word choice by each speaker. The anatomical regions identified in these analyses are presented in Tables 3, 4 and correspond to Figures 4, 5, respectively. These tables include the Montreal Neurological Institute, MNI, coordinates based on the TD ICBM MNI atlas (Maldjian et al., 2003) for each cluster in addition to statistical information related to the peak contrast including *t*-values with associated probability, p, and degrees of freedom, df. Brodmann's Area, BA, is associated with the identification of each anatomic region in the cluster including the probability of inclusion and a relative estimate of the cluster size (n of voxels).

**Table 3:** GLM contrast comparison: disagree [Talking > Listening] with ASE co-regressor, (deOxyHb signals).

**Contrast**	**Contrast threshold**	**Peak voxels**						**Anatomical regions in cluster**	**BA^***c***^**	**Probability**	**n of voxels**
		**MNI coordinates**^*******a*******^			***t*-value**	***p***	**Df^***b***^**				
[Talking > Listening]	*p =* 0.05	56	6	0	3.19	0.001	37	Superior temporal gyrus	22	0.41	1,158
(red)								Middle temporal gyrus	21	0.15	
								Temporopolar area	38	0.15	
Right hemisphere								Pars opercularis	44	0.13	
		12	58	36	3.00	0.002	37	Dorsolateral prefrontal cortex	9	0.56	85
								Frontopolar cortex	10	0.37	
	*p =* 0.05	−54	−2	6	3.55	0.001	37	Superior temporal gyrus	22	0.63	1,338
								Pre– and supplementary motor cortex	6	0.19	
								Middle temporal gyrus	21	0.10	
Left hemisphere		−60	−34	42	2.66	0.006	37	Supramarginal gyrus	40	0.73	81
								Primary somatosensory cortex	2	0.15	
		−52	−66	12	2.59	0.007	37	Extrastriate visual cortex (V3)	19	0.34	70
								Angular gyrus	39	0.34	
								Occipitotemporal cortex	37	0.15	
[Listening > Talking]	*p =* 0.05	70	−40	6	−2.73	0.005	37	Superior temporal gyrus	22	0.58	198
(blue)								Middle temporal gyrus	21	0.35	
		58	−52	38	−2.42	0.010	37	Supramarginal gyrus	40	0.87	153
Right Hemisphere								Angular gyrus	39	0.13	
Left Hemisphere	*p =* 0.05	−44	−42	50	−2.25	0.015	37	Supramarginal gyrus	40	0.98	93

a *Coordinates are based on the MNI system and (–) indicates left hemisphere*.

b *df, degrees of freedom*.

c *BA, Brodmann area identified by the TD ICBM MNI atlas (Maldjian et al., 2003) (see Figure 4)*.

**Table 4:** GLM contrast comparison: agree [Talking > Listening] with ASE co-regressor, (deOxyHb signals).

**Contrast**	**Contrast threshold**	**Peak voxels**						**Anatomical regions in cluster**	**BA^***c***^**	**Probability**	***n* of voxels**
		**MNI coordinates**^*******a*******^			***t*-value**	**p**	**Df^***b***^**				
[Talking > Listening]	*p =* 0.05	60	−10	−8	2.97	0.003	37	Middle temporal gyrus	21	0.92	510
(red)		60	−16	44	2.65	0.006	37	Pre– and supplementary motor cortex	6	0.41	209
								Primary somatosensory cortex	3	0.20	
Right hemisphere								Primary somatosensory cortex	1	0.18	
								Primary somatosensory cortex	2	0.13	
		40	−6	52	2.32	0.013	37	Pre- and supplementary motor cortex	6	0.94	94
	*p =* 0.05	−46	24	−4	2.53	0.008	37	Inferior frontal gyrus	47	0.67	700
								Temporopolar area	38	0.21	
		−14	58	14	2.03	0.025	37	Frontopolar cortex	10	1.00	151
		−52	−16	38	2.56	0.007	37	Pre- and supplementary motor cortex	6	0.63	122
Left hemisphere								Primary somatosensory cortex	3	0.20	
								Primary motor cortex	4	0.10	
		−40	18	46	2.33	0.013	37	Frontal eye fields	8	0.70	116
								Dorsolateral prefrontal cortex	9	0.16	
								Pre- and supplementary motor cortex	6	0.15	
[Listening>Talking]	*p =* 0.05	70	−44	0	−3.13	0.002	37	Middle temporal gyrus	21	0.52	430
(blue)								Superior temporal gyrus	22	0.37	
								Occipitotemporal cortex	37	0.11	
		48	44	16	−2.97	0.003	37	Dorsolateral prefrontal cortex	46	0.69	365
								frontopolar cortex	10	0.27	
Right hemisphere		52	18	36	−3.09	0.002	37	Dorsolateral prefrontal cortex	9	0.60	104
								Frontal eye fields	8	0.24	
								Dorsolateral prefrontal cortex	46	0.10	
		52	−68	26	−2.11	0.021	37	Angular gyrus	39	0.81	54
								Extrastriate visual cortex (V3)	19	0.15	
	*p =* 0.05	−48	30	28	−2.96	0.003	37	Dorsolateral prefrontal cortex	46	0.56	226
Left hemisphere								Dorsolateral prefrontal cortex	9	0.33	
								Pars triangularis	45	0.11	

a *Coordinates are based on the MNI system and (−) indicates left hemisphere*.

b *df, degrees of freedom*;

c *BA, Brodmann area identified by the TD ICBM MNI atlas (Maldjian et al., 2003) (see Figure 5)*.

#### Talking and Listening During Disagreement

Talking (relative to listening) (red) during disagreement (Figure 4) with a partner engages components of the well-known language system including left hemisphere (right panel) Wernicke's Area (AG, angular gyrus; SMG, supramarginal gyrus and Broca's Area (aSTG anterior superior temporal gyrus and components associated with the MC, motor cortex. These frontal systems are also observed bilaterally and include mSTG, middle superior temporal gyrus; ParsO, pars opercularis; DLPFC, dorsolateral prefrontal cortex; FP, frontopolar region. See Table 3, (top rows).

Co-activity of both bilateral frontal and left posterior regions associated with talking (relative to listening) during disagreement is consistent with modulation of this large-scale cortical network. Listening during disagreement (blue) engages regions in the right temporoparietal junction, TPJ, including angular gyrus, AG; superior temporal gyrus, STG; middle temporal gyrus, MTG; and bilateral supramarginal gyrus, SMG. See Table 3 (bottom rows). These right posterior regions, left panel, are part of the well-known social network (Carter and Huettel, 2013), and have been shown previously to be associated with live eye-to-eye contact (Noah et al., 2020).

#### Talking and Listening During Agreement

Talking (relative to listening) (red) during agreement (Figure 5) with a partner engages components of the frontal speech production system including left hemisphere (right panel) Broca's Area (inferior frontal gyrus, IFG; anterior superior temporal gyrus, STG; and components associated with the motor cortex, MC. See Table 4, (top rows). Findings are consistent with neural findings previously observed for live two-person talking (relative to listening) when the topic was a simple object naming and description (Hirsch et al., 2018). Listening during agreement (blue) engaged regions in the right temporoparietal junction, TPJ, including angular gyrus, AG; superior temporal gyrus, STG; middle temporal gyrus, MTG, consistent with finding for disagreement (above). See Table 4 (bottom rows).

#### Comparison of Disagreement and Agreement Conditions (GLM) During the Talking [Talking > Listening] Function

**Figure 6:**
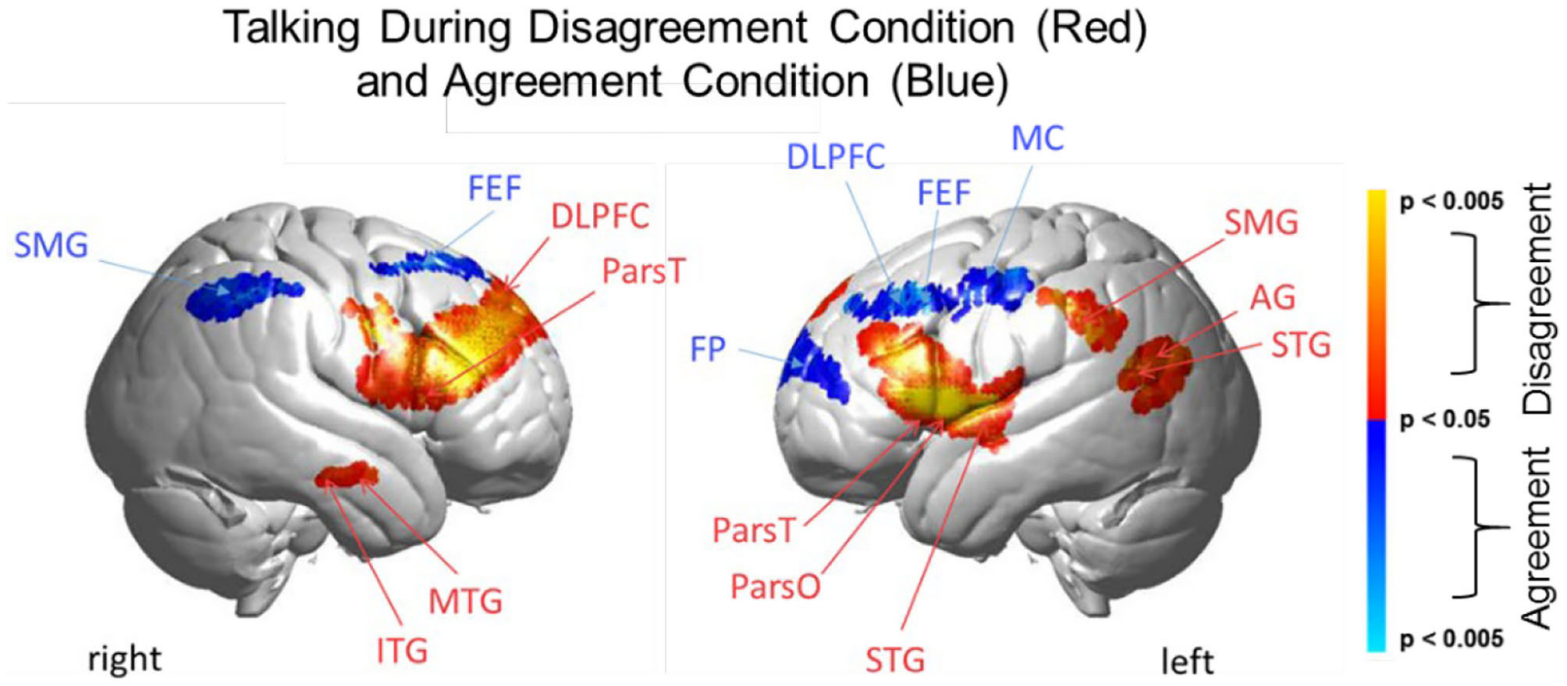
Talking condition. Brain activity clusters represent functions of Disagreement [talking > listening] (red) and Agreement [talking > listening] (blue). Neural systems most active during talking and disagreement conditions (red) include bilateral frontal regions [DLPFC, dorsolateral prefrontal cortex; ParsT, pars triangularis, ParsO, pars opercularis]. Left hemisphere SMG, supramarginal gyrus; AG, angular gyrus; STG, superior temporal gyrus are also included. Neural systems most active during talking and agreement conditions (blue) include bilateral FEF, frontal eye fields right SMG, supramarginal gyrus and left FP, frontopolar activity. See Table 5.

The above analyses that compare talking (relative to listening) and listening (relative to talking) for each condition (disagreement and agreement) separately suggest, as hypothesized, that the neural correlates underlying verbal language exchanges in real time adapt to social situations such as whether the discourse is one of agreement or disagreement. In order to directly compare the two conditions, disagreement and agreement, Figure 6 shows [talking > listening] for [disagreement > agreement] (red) and [agreement > disagreement] (blue). The findings illustrated by the heat maps (Figures 4, 5) and associated Statistical Tables (Tables 3, 4, top rows) are consistent with this hypothesis. Talking under conditions of disagreement (relative to agreement) shows increased activity in bilateral frontal regions: DLPFC, dorsolateral prefrontal cortex; ParsT, pars triangularis; ParsO, pars opercularis; left posterior regions including SMG, supramarginal gyrus; AG, angular gyrus; STG, superior temporal gyrus.

**Table 5:** GLM contrast comparison: talking [Disagree > Agree] with ASE co-regressor, (deOxyHb signals).

**Contrast**	**Contrast threshold**	**Peak voxels**						**Anatomical regions in cluster**	**BA^**c**^**	**Probability**	**n of voxels**
		**MNI Coordinates**^****a****^			***t*-value**	**p**	**df^**b**^**				
[Disagree > Agree]	*p =* 0.05	52	32	20	4.07	0.000	37	Dorsolateral prefrontal cortex	46	0.69	2,003
(red)								Pars triangularis	45	0.27	
		60	−12	−18	1.98	0.027	37	Middle temporal gyrus	21	0.61	62
Right hemisphere								Inferior temporal gyrus	20	0.39	
	*p =* 0.05	−52	10	10	3.28	0.001	37	Pars opercularis	44	0.40	1,193
								Superior temporal gyrus	22	0.25	
								Pars triangularis	45	0.19	
								Pre- and supplementary motor cortex	6	0.12	
		−58	−58	18	2.35	0.012	37	angular gyrus	39	0.37	167
Left hemisphere								Superior temporal gyrus	22	0.30	
								Supramarginal gyrus	40	0.13	
								Middle temporal gyrus	21	0.11	
		−62	−36	40	2.52	0.008	37	Supramarginal gyrus	40	0.81	60
								Primary somatosensory cortex	2	0.11	
[Agree>Disagree]	*p =* 0.05	58	−42	46	−2.53	0.008	37	Supramarginal gyrus	40	0.97	120
(blue)		28	24	52	−2.43	0.010	37	Frontal eye fields	8	0.68	96
Right hemisphere								Pre- and supplementary motor cortex	6	0.32	
	*p =* 0.05	−46	−14	46	−2.34	0.012	37	Pre- and supplementary motor cortex	6	0.44	123
								Primary somatosensory cortex	3	0.32	
								Primary motor cortex	4	0.17	
Left hemisphere		−6	58	18	−2.14	0.019	37	Frontopolar cortex	10	1.00	97
		−44	18	44	−2.67	0.006	37	Frontal eye fields	8	0.53	79
								Dorsolateral prefrontal cortex	9	0.34	
								Pre- and supplementary motor cortex	6	0.13	

a *Coordinates are based on the MNI system and (−) indicates left hemisphere*.

b *df, degrees of freedom*;

c *BA, Brodmann area identified by the TD ICBM MNI atlas (Maldjian et al., 2003) (See Figure 6)*.

#### Cross-Brain Coherence During Disagree and Agree Conditions

**Figure 7:**
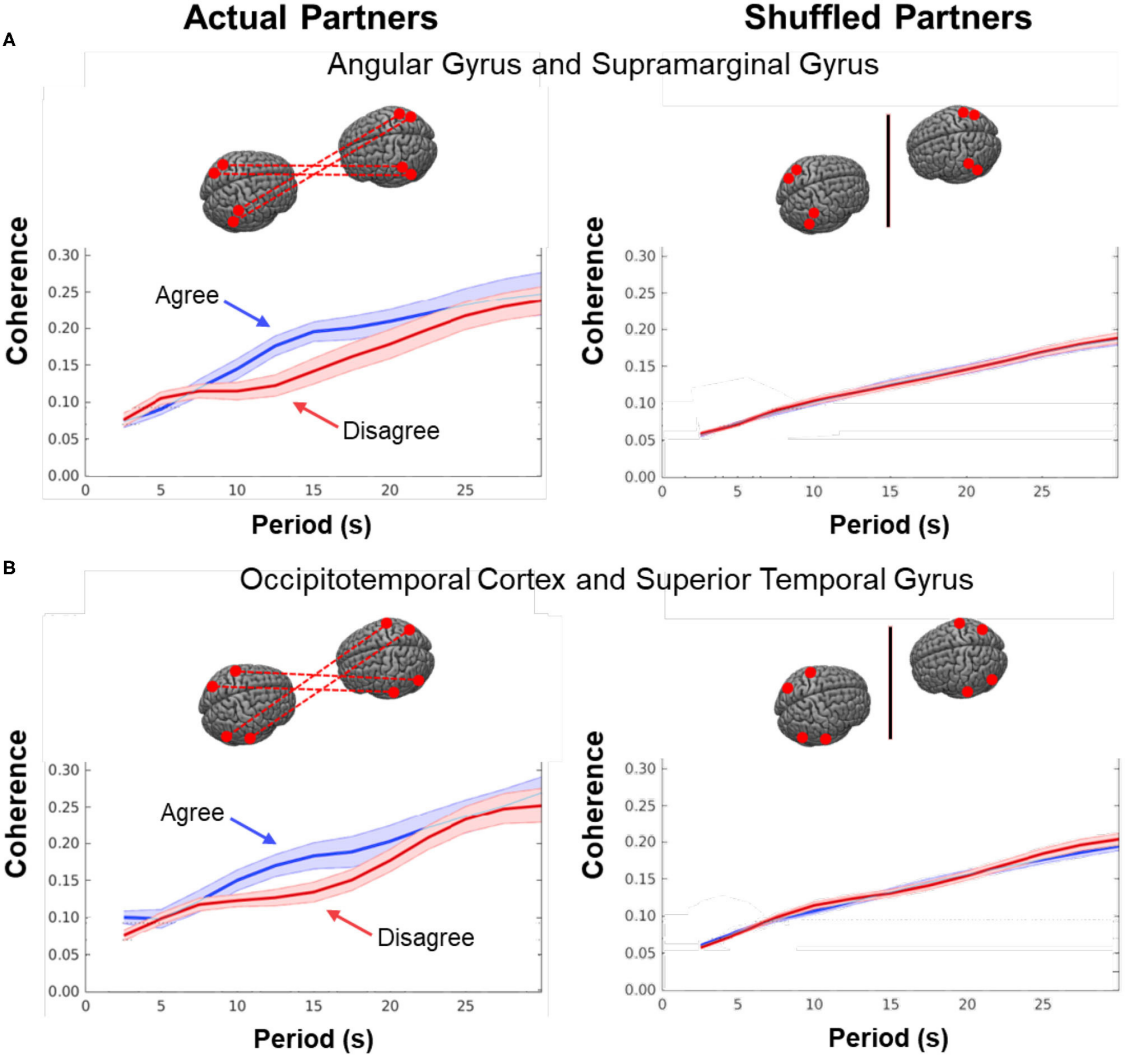
Cross-brain coherence. Signal coherence between dyads (y-axis) is plotted against the period (x-axis) for the Disagree (red) and Agree (blue) conditions (shaded areas: ±1 SEM). The left column shows coherence between actual partners, and the right column shows coherence between shuffled partners. Greater signal coherence was observed between actual partners in **(A)** angular gyrus to supramarginal gyrus and **(B)** occipitotemporal cortex to superior temporal gyrus. In contrast, no significant differences were found in coherence between shuffled partners during either condition.

As noted above, dynamic neural coupling has been proposed as a neural process that represents sharing of information between dyads during live interactions (Hasson et al., 2004, 2008; Dumas et al., 2010; Hasson and Frith, 2016; R Core Team, 2018). This paradigm provides an opportunity to further develop this theoretical framework by exploring the variations in neural coupling that occur in relation to the behavioral conditions of dialogue with agreement and disagreement. Here we apply a measure of neural coupling as a dyadic property that is compared during conditions of agreement and disagreement. Neural coupling within the interacting dyads is quantified by cross-brain synchrony of hemodynamic signals, i.e., coherence (shown in Figure 7). The correlation between the decomposed wavelet signals of the two partners (y-axis) is plotted against the wavelengths, i.e., periods, in secs (x-axis) of the wavelets originating from the decomposed neural signals (Zhang et al., 2020). The coherence function for agreement is shown in blue and in red for disagreement. These functions represent dyadic activity where two tasks, i.e., talking and listening, occur simultaneously during the experimental sessions. Cross-brain coherence was greater for the agreement conditions than the disagreement conditions for two regional sources of signals: angular gyrus to supramarginal gyrus (Figure 7A) and occipitotemporal cortex to superior temporal gyrus (Figure 7B). Specifically, this increase was observed between 10 and 16 secs (*p* < 0.01 (A) and *p* < 0.05 (B) during the experimental cycle of 30 s, i.e., 15 s per epoch where a full cycle of two epochs consisted of talking (15 s) and listening (15 s). Note these effect sizes are not corrected for multiple comparisons. To further evaluate the neural coupling, real partners were compared to computationally shuffled partners. If the hypothesis of neural coupling is supported, then it would be expected that the real partners where behavioral and social coupling occurred would show a greater effect than the shuffled conditions where the reciprocal social interactions did not occur. The observed null findings of the shuffled cases compared to the real cases suggests that the real social interactions are also synchronous. There was no difference between the coherence functions in the case of the shuffled partners (Figure 7, right column), which is consistent with the interpretation that the coherence was related to the synchronous exchange of dynamic social cues specific to the partners. Further, given the above theoretical framework, this finding also suggests that the interchange of these social cues increased during agreement dialogue relative to disagreement.

In contrast, neural systems most active during talking while in agreement (relative to disagreement), blue, include bilateral frontal eye fields, FEF, right supramarginal gyrus, SMG, and left frontopolar, FP, activity. See Tables 3, 4, bottom rows.

## Discussion

While agreement and disagreement arise naturally within dyadic interactions, behavioral observation in this investigation is facilitated by a partially structured experimental paradigm. Here we set up a “debate-like” situation where the opinions of the participating individuals about a given topic are either in alignment or not. The two-person paradigm employed here provides an opportunity for participants to engage in a face-to-face unscripted discourse expressing either different or similar points of view with the dialectical intent to engage in reasoned arguments or discussion in a structured format. As in the case of a formal debate, the talking epochs are timed so that each partner has equal time for discussion or rebuttal. This task is intended to establish dyadic states that have previously been described as either *dialectical misattunement* where the unfolding of an interaction over time results in increasingly divergent interaction styles, or *dialectical attunement* where the unfolding of an interaction over time results in increasingly convergent interaction styles (Bolis et al., 2017). The dyads are assumed to have differed on measures not considered here. The nature of “natural experiments” includes absence of experimental control over the specific dialogues, emotional expressions, and social interactions with the partner. Nonetheless, we provide three sources of behavioral evidence that the dialogues overall represented either states of dyadic agreement or disagreement consistent with the conditions that were investigated in this experiment. These sources include: (1) confirmation by independent raters that the interlocutors actually engaged in the assigned topic for the dialogue. Their personal opinions were known from an *a priori* survey to be either mutually aligned (agreement) or divergent (disagreement); (2) documentation by word feature analysis that the relative frequency of key words representative of either agreement or disagreement were consistent with the condition type; and (3) finally, statistical evidence consistent with the finding that the acoustical properties of the recorded narratives including syllable rate, SYLR; fundamental frequency range, RF0; median fundamental frequency, MF0; and area under the speech envelope, ASE, were different, and indeed, were consistently elevated for disagreement dialogues. Here we aim to compare the neural interplay between dyads during these two conditions: (1) where the individual interlocutors are engaged in an interaction with “misattuned,” i.e., incongruent opinions (disagreement); and (2) where the same individual interlocutors are engaged in an interaction with “attuned,” i.e., congruent opinions (agreement). Findings are related to the dyadic unit as a theoretical frame for the establishment of neural coupling as a component of social interactions.

Human listeners are known to be exquisitely capable of assessing emotional content and degree of arousal in speech, even from unknown languages, and acoustic correlates of emotion have been well-studied (e.g., Williams and Stevens, 1972). Although “disagreement” can be expressed on many levels (choice of vocabulary, syntactic constructions, etc.), its characteristic prosody (*inter alia*, extended fundamental frequency range, greater acoustic energy) is similar to patterns also associated with anger, and recent machine learning approaches have exploited these measures to automatically identify disagreement “hot spots” in conversation (e.g., Wrede and Shriberg, 2003). In this study the effects of acoustic variation thus provide a behavioral validation of the expected dyadic conditions of agreement and disagreement.

Findings validate the well-established canonical language related regions that are activated during the two-person speaking and listening paradigm (Figure 2A). This provides a foundation for further interpretation. Audio behavioral measures show that the acoustical properties of the recorded speech, including the fundamental frequency range, median fundamental frequency, syllable rate, and acoustic energy were elevated during disagreement conditions relative to agreement conditions (Figure 3). Here we evaluate the neural modulations in response to these conditions. One possible model based on the assumption of a modular functional architecture, *functional specificity*, proposes that modulations will be localized to the canonical language regions. An alternative model, the *multiple networks* model, predicts that verbal agreement and disagreement will engage distinctive combinations of language-related cognitive and perceptual networks.

### Talking and Listening During Disagreement

Talking during disagreement (Figure 4, red) showed the involvement of the frontoparietal network including DLPFC (right hemisphere), parietal (supramarginal gyrus), and visual attentional systems (angular gyrus, occipitotemporal cortex (left hemisphere). Listening during disagreement (Figure 4, blue) showed the additional involvement of the social network including activity in the right temporal-parietal junction (middle and superior temporal gyrus and angular gyrus) as well as bilateral supramarginal gyrus. Together, talking and listening during disagreement resulted in up-regulation of the frontoparietal network, the visual attention system, and the social network consistent with the multiple networks model.

### Talking and Listening During Agreement

Talking during agreement (Figure 5, red) was associated with the expected pattern of activity based on speech production (Broca's Area), natural eye movements (frontal eye fields), and left hemisphere frontal systems. Listening during agreement (blue) showed the additional involvement of the right hemisphere social network including activity in the right temporal-parietal junction (middle and superior temporal gyrus and angular gyrus) as well as the high-level visual attention system (V3 and the occipitotemporal cortex), and the frontal executive and attention systems including bilateral DLPFC and the left frontopolar regions. Together, talking and listening during agreement also resulted in up-regulation of the frontoparietal network, the visual attention system, and the social network consistent with the multiple networks model.

### Talking During Disagreement and Agreement

Direct comparison of these intermixed neural systems during disagreement (Figure 6, red) and agreement (blue) during talking [talking > listening] reveals how these networks are modulated during these two attunement situations. We show the comparison for talking rather than for listening because spoken language, in contrast to receptive language, is observable and can be related to the acoustic analysis. Receptive processes such as listening are subjective and therefore not explicitly quantifiable in this paradigm. The behavioral measures of acoustical properties of the spoken narratives predict a difference between these two conditions. Consistent with this prediction, activity was increased in the frontoparietal network (bilateral DLPFC, left supramarginal, angular, and superior temporal gyri) during disagreement (relative to agreement). In contrast, talking during agreement (blue) modulated a component of the right hemisphere social system (supramarginal gyrus) including the visual attention system and bilateral frontal eye-fields, left DLPFC, and frontopolar regions. These comparisons illustrate large scale variations in language, cognitive, executive, social, and visual processes depending upon the social context related to the attunement or misattunement of opinions with another individual. Together these findings are consistent with the hypothesis that neural systems associated with dyadic states of verbal disagreement and agreement are differentiable, and further suggest a role for the DLPFC in the adaptive neural mechanism, especially for disagreement.

### A Theoretical Shift From the Single Brain to the Dyad

The development of two-brain functional imaging systems enables the paradigm shift from a frame of reference focused on the single brain to a frame of reference focused on the interactive human dyad. This shift includes computational approaches that model the dyadic unit and extend methods for quantifying synchronous systems across brains. Over the last decade, observations of neural coupling during interactive tasks have become a cornerstone for an emerging theoretical framework of dynamic cross-brain neural processes (Saito et al., 2010; Schippers et al., 2010; Funane et al., 2011; Cui et al., 2012; Dommer et al., 2012; Holper et al., 2012; Konvalinka and Roepstorff, 2012; Tanabe et al., 2012; Kawasaki et al., 2013; Schilbach et al., 2013; Scholkmann et al., 2013a; Osaka et al., 2014, 2015; Jiang et al., 2015; Koike et al., 2016; Liu et al., 2016, 2017; Tang et al., 2016; Dravida et al., 2017; Hirsch et al., 2017, 2018; Kingsbury et al., 2019; Zhang and Yartsev, 2019; Descorbeth et al., 2020; Noah et al., 2020).

A recent investigation of neural coupling with known temporal inputs to the visual system provided by frequencies of reversing checkerboard stimuli strengthens the evidence in support of this approach (Zhang et al., 2020). In this prior study, neural coupling, as represented by wavelet coherence of hemodynamic signals, was compared to the known sequences of visual stimulation using fNIRS. The coherence of input and known frequencies was consistent with the coherence of the predicted and observed fNIRS signals. This objective evidence that coherence between brains can be predicted from known (simulated) signal coherence based on input sequences further validates wavelet analysis as a measure of neural coupling and as a computational approach for investigation of the neural mechanisms that underlie behavioral attunement. However, this technique and the experimental paradigm is relatively exploratory. The coherence analysis remains limited, in part, by the lack of phase information between the two partners. Factors that contribute to the absence of a detectable latency include the presence of random noise in the data, individual differences in hemodynamic response functions, and use of the residual signals which are not task generated. A latency value that is not different from zero can be interpreted as consistent with interactions over time where partners vary between leading and following which is likely in the case where listening and talking are alternated. Further, the spatial resolution of the acquired fNIRS signals is relatively coarse (~3 cm) and therefore source localizations of the origins of the coherent signals are also similarly limited.

This dyadic frame of reference including the two-person experimental paradigm and accompanying computational tools, provides a general platform for investigation of the neural systems that underlie brain functions during natural discourse where partners express opinions that are either congruent or discordant. Neural coupling (Figure 7), as quantified by mutual coherence, was greater during agreement than disagreement and included cortical regions previously associated with live face-to-face interaction, i.e., angular to supramarginal gyri, and fusiform (occipitotemporal cortex) to superior temporal gyrus. The regional locations of these synchronous cross-brain signals were also consistent with the activity observed during listening when in agreement (Figure 5, blue). However, coupled neural activity, as represented here by the hemodynamic signal, includes both the listener and the speaker (non-symmetrical neural coupling, Hasson and Frith, 2016), and shows a synchronous temporal hemodynamic pattern in the range of wavelengths (periods) of 10–16 s. Prior models of information sharing (Stephens et al., 2010; Hasson and Frith, 2016) are not sufficiently developed to predict these findings. Although coherence can be interpreted as a mechanism by which live social information is shared between the receiver and sender, current models of neural coupling do not predict which condition, agreement or disagreement, would result in higher cross-brain coherence. Further, the current state of the framework does not predict which regions of brain would be most coherent.

The putative underlying mechanism for neural coherence could possibly involve differentiable patterns of eye-to-eye contact between interlocutors, and this hypothesis is a target for future research. The cognitive and perceptual significance of these coherence effects is yet to be determined, but preliminary work has shown that they predictably influence the quality of communication and comprehension (Stephens et al., 2010; Hasson et al., 2012; Hasson and Frith, 2016), arousal and social judgments (Vanutelli et al., 2017; Descorbeth et al., 2020), verbal interaction (Hirsch et al., 2018), eye contact (Noah et al., 2020) and decision making (Cui et al., 2012).

### Convergence

When humans interact they typically show converging patterns of coordinated behavior, as in walking in step or synchronized applause. In the complex interplay of dyadic conversation, numerous studies have shown that over time participants tend to accommodate their speech patterns to one another (Pardo, 2006; Babel, 2012); alternatively they may also diverge from one another under conditions of disagreement or dislike (Bourhis and Giles, 1977). Neural correlates of these behavioral patterns of accommodation, or *phonetic convergence*, have also been proposed (Garnier et al., 2013) and are active areas of investigation. While our acoustic measures clearly show differences in behavioral results indicating enhanced speaker emphasis and engagement for the disagreement condition *vs*. the agreement condition, we did not find consistent evidence for phonetic convergence as might be predicted by the neural coherence overall, although some individual dyads showed such effects. We speculate that the somewhat artificial nature of the 15 s turns imposed by the protocol (to support the block design) may have disrupted these (typically subtle) acoustic effects.

### Summary and Conclusion

Verbal face-to-face communication is central to everyday transactions between humans throughout the entire lifespan. The foundational nature of live and interactive social functions is in contrast to the nascent development of neurocognitive theories to explain them which have been limited by lack of data from live and natural social interactions. Here we have addressed this knowledge gap by recording neural responses and speech concurrently during live face-to-face discussions of topics where the interlocutors were either in agreement or disagreement, a common feature characteristic of verbal exchanges between two individuals. The dialectical misattunement hypothesis (Bolis et al., 2017) proposes that disturbances in the reciprocal unfolding of an interaction result in a change of the dyadic state, thus predicting a behavioral and neural difference between the two conditions employed in this study. Findings in this investigation are consistent with this prediction. A theoretical neural framework for the effects observed in this investigation remain nascent but contribute to an emerging neuroscience of social interactions that may serve to coordinate activities of multiple component systems often investigated alone such as face processing, language production, and theory of mind. In so doing, this paradigm provides a template for developing methods and models to access neural processes that are responsive to spontaneous, rapid, and multimodal components of live interactions.

## Data Availability Statement

The datasets presented in this study can be found in online repositories. The names of the repository/repositories and accession number(s) can be found below: www.fmri.org.

## Ethics Statement

The studies involving human participants were reviewed and approved by Yale Human Research Protection Program. The patients/participants provided their written informed consent to participate in this study.

## Author Contributions

JH contributed to the design, data collection, analysis, and manuscript preparation. MT performed the acoustic analyses and contributed to manuscript preparation. JN contributed to all aspects of the study with specific responsibility for signal acquisition. XZ also contributed to all aspects of the study with specific responsibility for data analysis. AS-M ran the experiment, collected the SurveyMonkey questionnaires, selected the dyads, and performed the initial data analyses. MB contributed useful discussion and overview throughout the project. All authors contributed to manuscript revision, read, and approved the submitted version.

## Conflict of Interest

The authors declare that the research was conducted in the absence of any commercial or financial relationships that could be construed as a potential conflict of interest.

## References

[B1] Babel M. (2012). Evidence for phonetic and social selectivity in spontaneous phonetic imitation. J. Phon. 40, 177–189. 10.1016/j.wocn.2011.09.001

[B2] Babiloni F. Astolfi L. (2014). Social neuroscience and hyperscanning techniques: past, present and future. Neurosci. Biobeh. Rev. 44, 76–93. 10.1016/j.neubiorev.2012.07.006 PMC3522775 22917915

[B3] Bates D. Mächler M. Bolker B. Walker S. (2015). Fitting linear mixed-effects models using *lme4*. J. Stat. Softw. 67, 1–48. 10.18637/jss.v067.i01

[B4] Binder J. R. Frost J. A. Hammeke T. A. Bellgowan P. S. Springer J. A. Kaufman J. N.. (2000). Human temporal lobe activation by speech and nonspeech sounds. Cerebral Cortex 10, 512–528. 10.1093/cercor/10.5.512 10847601

[B5] Boas D. A. Dale A. M. Franceschini M. A. (2004). Diffuse optical imaging of brain activation: approaches to optimizing image sensitivity, resolution, and accuracy. NeuroImage 23 (Suppl. 1), S275–S288. 10.1016/j.neuroimage.2004.07.011 15501097

[B6] Boas D. A. Elwell C. E. Ferrari M. Taga G. (2014). Twenty years of functional near-infrared spectroscopy: Introduction for the special issue. NeuroImage 85(Pt 1), 1–5. 10.1016/j.neuroimage.2013.11.033 24321364

[B7] Boersma P. (1993). Accurate short-term analysis of the fundamental frequency and the harmonics-to-noise ratio of a sampled sound. Proc. Instit. Phonetic Sci. 17, 97–110.

[B8] Bolis D. Balsters J. Wenderoth N. Becchio C. Schilbach L. (2017). Beyond autism: Introducing the dialectical misattunement hypothesis and a bayesian account of intersubjectivity. Psychopathology 50, 355–372. 10.1159/000484353 29232684

[B9] Bourhis R. Y. Giles H. (1977). The language of intergroup distinctiveness. Lang. Ethn. Intergr. Relat. 13:119.

[B10] Carter R. M. Huettel S. A. (2013). A nexus model of the temporal–parietal junction. Trends Cogn. Sci. 17, 328–336. 10.1016/j.tics.2013.05.007 23790322 PMC3750983

[B11] Cavallo A. Lungu O. Becchio C. Ansuini C. Rustichini A. Fadiga L. (2015). When gaze opens the channel for communication: integrative role of IFG and MPFC. NeuroImage 119, 63–69. 10.1016/j.neuroimage.2015.06.025 26080312

[B12] Chang L. Tsao D. Y. (2017). The code for facial identity in the primate brain. Cell 169, 1013–1028. 10.1016/j.cell.2017.05.011 28575666 PMC8088389

[B13] Cui X. Bray S. Bryant D. M. Glover G. H. Reiss A. L. (2011). A quantitative comparison of NIRS and fMRI across multiple cognitive tasks. NeuroImage 54, 2808–2821. 10.1016/j.neuroimage.2010.10.069 21047559 PMC3021967

[B14] Cui X. Bryant D. M. Reiss A. L. (2012). NIRS-based hyperscanning reveals increased interpersonal coherence in superior frontal cortex during cooperation. NeuroImage 59, 2430–2437. 10.1016/j.neuroimage.2011.09.003 21933717 PMC3254802

[B15] Descorbeth O. Zhang X. Noah J. A. Hirsch J. (2020). Neural processes for live pro-social dialogue between dyads with socioeconomic disparity. Soc. Cogn. Affect. Neurosci. 15, 875–887. 10.1093/scan/nsaa120 32879986 PMC7543936

[B16] Dommer L. Jäger N. Scholkmann F. Wolf M. Holper L. (2012). Between-brain coherence during joint n-back task performance: a two-person functional near-infrared spectroscopy study. Behav. Brain Res. 234, 212–222. 10.1016/j.bbr.2012.06.024 22750679

[B17] Dravida S. Noah J. A. Zhang X. Hirsch J. (2017). Comparison of oxyhemoglobin and deoxyhemoglobin signal reliability with and without global mean removal for digit manipulation motor tasks. Neurophotonics 5:011006. 10.1117/1.NPh.5.1.011006 28924566 PMC5597778

[B18] Dravida S. Noah J. A. Zhang X. Hirsch J. (2020). Joint attention during live person-to-person contact activates rTPJ, including a sub-component associated with spontaneous eye-to-eye contact. Front. Hum. Neurosci. 14, 1–6. 10.3389/fnhum.2020.00201 32581746 PMC7283505

[B19] Duchaine B. Yovel G. (2015). A revised neural framework for face processing. Annu. Rev. Vis. Sci. 1, 393–416. 10.1146/annurev-vision-082114-035518 28532371

[B20] Dumas G. Nadel J. Soussignan R. Martinerie J. Garnero L. (2010). Inter-brain synchronization during social interaction. PLoS ONE 5:e12166. 10.1371/journal.pone.0012166 20808907 PMC2923151

[B21] Eggebrecht A. T. White B. R. Ferradal S. L. Chen C. Zhan Y. Snyder A. Z.. (2012). A quantitative spatial comparison of high-density diffuse optical tomography and fMRI cortical mapping. NeuroImage 61, 1120–1128. 10.1016/j.neuroimage.2012.01.124 22330315 PMC3581336

[B22] Ferradal S. L. Eggebrecht A. T. Hassanpour M. Snyder A. Z. Culver J. P. (2014). Atlas-based head modeling and spatial normalization for high-density diffuse optical tomography: in vivo validation against fMRI. NeuroImage 85, 117–126. 10.1016/j.neuroimage.2013.03.069 23578579 PMC4433751

[B23] Ferrari M. Quaresima V. (2012). A brief review on the history of human functional near-infrared spectroscopy. (fNIRS). development and fields of application. NeuroImage 63, 921–935. 10.1016/j.neuroimage.2012.03.049 22510258

[B24] Friston K. J. (1994). Functional and effective connectivity in neuroimaging: A synthesis. Hum. Brain Map. 2, 56–78. 10.1002/hbm.460020107

[B25] Friston K. J. Harrison L. Penny W. (2003). Dynamic causal modelling. NeuroImage 19, 1273–1302. 10.1016/S1053-8119(03)00202-7 12948688

[B26] Funane T. Kiguchi M. Atsumori H. Sato H. Kubota K. Koizumi H. (2011). Synchronous activity of two people's prefrontal cortices during a cooperative task measured by simultaneous near-infrared spectroscopy. J. Biomed. Optics 16:077011. 10.1117/1.3602853 21806291

[B27] Gabrieli J. D. Poldrack R. A. Desmond J. E. (1998). The role of left prefrontal cortex in language and memory. Proc. Natl. Acad. Sci. U.S.A. 95, 906–913. 10.1073/pnas.95.3.906 9448258 PMC33815

[B28] Galley M. McKeown K. Hirschberg J. Shriberg E. (2004). “Identifying agreement and disagreement in conversational speech: use of Bayesian networks to model pragmatic dependencies,” in ACL '04: Proceedings of the 42nd Annual Meeting on Association for Computational Linguistics (Barcelona), 669 10.3115/1218955.1219040

[B29] García A. M. Ibáñez A. (2014). Two-person neuroscience and naturalistic social communication: the role of language and linguistic variables in brain-coupling research. Front. Psychiatry 5:124. 10.3389/fpsyt.2014.00124 25249986 PMC4155792

[B30] Garnier M. Lamalle L. Sato M. (2013). Neural correlates of phonetic convergence and speech imitation. Front. Psychol. 4:600. 10.3389/fpsyg.2013.00600 24062704 PMC3769680

[B31] George N. Driver J. Dolan R. J. (2001). Seen gaze-direction modulates fusiform activity and its coupling with other brain areas during face processing. NeuroImage 13, 1102–1112. 10.1006/nimg.2001.0769 11352615

[B32] Germesin S. Wilson T. (2009). “Agreement Detection in Multiparty Conversation,” in ICMI-MLMI '09: Proceedings of the 2009 International Conference on Multimodal Interfaces (Cambridge, MA), 7–14. 10.1145/1647314.1647319

[B33] Gracco V. L. Tremblay P. Pike B. (2005). Imaging speech production using fMRI. Neuroimage 26, 294–301. 10.1016/j.neuroimage.2005.01.033 15862230

[B34] Gregory S. W. Hoyt B. R. (1982). Conversation partner mutual adaptation as demonstrated by Fourier series analysis. J. Psychol. Res. 11, 35–46. 10.1007/BF01067500

[B35] Guenther F. H. (1995). Speech sound acquisition, coarticulation, and rate effects in a neural network model of speech production. Psychol. Rev. 102:594. 10.1037/0033-295X.102.3.594 7624456

[B36] Hagoort P. (2014). Nodes and networks in the neural architecture for language: Broca's region and beyond. Curr. Opin. Neurob. 28, 136–141. 10.1016/j.conb.2014.07.013 25062474

[B37] Hasson U. Frith C. D. (2016). Mirroring and beyond: Coupled dynamics as a generalized framework for modelling social interactions. Philosoph. Trans. R. Soc. B Biol. Sci. 371:20150366. 10.1098/rstb.2015.0366 27069044 PMC4843605

[B38] Hasson U. Ghazanfar A. A. Galantucci B. Garrod S. Keysers C. (2012). Brain-to-brain coupling: a mechanism for creating and sharing a social world. Trends Cogn. Sci. 16, 114–121. 10.1016/j.tics.2011.12.007 22221820 PMC3269540

[B39] Hasson U. Nir Y. Levy I. Fuhrmann G. Malach R. (2004). Intersubject synchronization of cortical activity during natural vision. Science 303, 1634–1640. 10.1126/science.1089506 15016991

[B40] Hasson U. Yang E. Vallines I. Heeger D. J. Rubin N. (2008). A hierarchy of temporal receptive windows in human cortex. J. Neurosci. 28, 2539–2550. 10.1523/JNEUROSCI.5487-07.2008 18322098 PMC2556707

[B41] Haxby J. V. Hoffman E. A. Gobbini M. I. (2000). The distributed human neural system for face perception. Trends Cogn. Sci. 4, 223–233. 10.1016/S1364-6613(00)01482-0 10827445

[B42] Hickok G. Poeppel D. (2000). Towards a functional neuroanatomy of speech perception. Trends Cogn. Sci. 4, 131–138. 10.1016/S1364-6613(00)01463-7 10740277

[B43] Hillard D. Ostendorf M. Shriberg E. (2003). “Detection of agreement vs. disagreement in meetings: training with unlabeled data,” in: Proceedings of the 2003 Conference of the North American Chapter of the Association for Computational Linguistics on Human Language Technology: Companion volume of the Proceedings of HLT-NAACL 2003 (Edmonton). 10.3115/1073483.1073495

[B44] Hirsch J. Noah J. A Zhang X. Dravida S. Ono Y. (2018). A cross-brain neural mechanism for human-to-human verbal communication. Soc. Cogn. Affect. Neurosci. 13, 907–920. 10.1093/scan/nsy070 30137601 PMC6137318

[B45] Hirsch J. Zhang X. Noah J. A. Ono Y. (2017). Frontal temporal and parietal systems synchronize within and across brains during live eye-to-eye contact. NeuroImage 157, 314–330. 10.1016/j.neuroimage.2017.06.018 28619652 PMC5863547

[B46] Hoffman E. A. Haxby J. V. (2000). Distinct representations of eye gaze and identity in the distributed human neural system for face perception. Nat. Neurosci. 3, 80–84. 10.1038/71152 10607399

[B47] Holper L. Scholkmann F. Wolf M. (2012). Between-brain connectivity during imitation measured by fNIRS. NeuroImage 63, 212–222. 10.1016/j.neuroimage.2012.06.028 22732563

[B48] Hooker C. I. Paller K. A. Gitelman D. R. Parrish T. B. Mesulam M.-M Reber P. J. (2003). Brain networks for analyzing eye gaze. Cogn. Brain Res. 17, 406–418. 10.1016/S0926-6410(03)00143-5 12880911 PMC4346170

[B49] Jiang J. Borowiak K. Tudge L. Otto C. von Kriegstein K. (2017). Neural mechanisms of eye contact when listening to another person talking. Soc Cogn. Affect. Neurosci. 12, 319–328. 10.1093/scan/nsw127 27576745 PMC5390711

[B50] Jiang J. Chen C. Dai B. Shi G. Ding G. Liu L.. (2015). Leader emergence through interpersonal neural synchronization. Proc. Natl. Acad. Sci. U.S.A. 112, 4274–4279. 10.1073/pnas.1422930112 25831535 PMC4394311

[B51] Jiang J. Dai B. Peng D. Zhu C. Liu L. Lu C. (2012). Neural synchronization during face-to-face communication. J. Neurosci. 32, 16064–16069. 10.1523/JNEUROSCI.2926-12.2012 23136442 PMC6621612

[B52] Kawasaki M. Yamada Y. Ushiku Y. Miyauchi E. Yamaguchi Y. (2013). Inter-brain synchronization during coordination of speech rhythm in human-to-human social interaction. Scient. Rep. 3:1692. 10.1038/srep01692 23603749 PMC3631767

[B53] Kingsbury L. Huang S. Wang J. Gu K. Golshani P. Wu Y. E.. (2019). Correlated neural activity and encoding of behavior across brains of socially interacting animals. Cell 178, 429–46.e416. 10.1016/j.cell.2019.05.022 31230711 PMC6625832

[B54] Kirilina E. Jelzow A. Heine A. Niessing M. Wabnitz H. Bruhl R.. (2012). The physiological origin of task-evoked systemic artefacts in functional near infrared spectroscopy. NeuroImage 61, 70–81. 10.1016/j.neuroimage.2012.02.074 22426347 PMC3348501

[B55] Koike T. Tanabe H. C. Adachi-Abe S. Okazaki S. Nakagawa E. Sasaki A.T.. (2019). Role of the right anterior insular cortex in joint attention-related identification with a partner. Soc. Cogn. Affect. Neurosci. 14, 1131–1145. 10.1093/scan/nsz087 31919530 PMC6970150

[B56] Koike T. Tanabe H. C. Okazaki S. Nakagawa E. Sasaki A. T. Shimada K.. (2016). Neural substrates of shared attention as social memory: A hyperscanning functional magnetic resonance imaging study. Neuroimage 125, 401–412. 10.1016/j.neuroimage.2015.09.076 26514295

[B57] Konvalinka I. Roepstorff A. (2012). The two-brain approach: How can mutually interacting brains teach us something about social interaction? Front. Hum. Neurosci. 6:215. 10.3389/fnhum.2012.00215 22837744 PMC3402900

[B58] Koster-Hale J. Saxe R. (2013). Theory of mind: a neural prediction problem. Neuron 79, 836–848. 10.1016/j.neuron.2013.08.020 24012000 PMC4041537

[B59] Kuznetsova A. Brockhoff P. B. Christensen R. H. (2017). *lmerTest* package: tests in linear mixed effects models. J. Stat. Softw. 82, 1–26. 10.18637/jss.v082.i13

[B60] Lachat F. Conty L. Hugueville L. George N. (2012). Gaze cueing effect in a face-to-face situation. J. Nonverbal Behav. 36, 177–190. 10.1007/s10919-012-0133-x

[B61] Levelt W. J. M. (1989). ACL-MIT Press Series in Natural-Language Processing. Speaking: From intention to articulation. The MIT Press.

[B62] Lindquist K. A. Barrett L. F. (2012). A functional architecture of the human brain: emerging insights from the science of emotion. Trends Cogn. Sci. 16, 533–540. 10.1016/j.tics.2012.09.005 23036719 PMC3482298

[B63] Liu N. Mok C. Witt E. E. Pradhan A. H. Chen J. E. Reiss A. L. (2016). NIRS-based hyperscanning reveals inter-brain neural synchronization during cooperative Jenga game with face-to-face communication. Front. Hum. Neurosci. 10:11. 10.3389/fnhum.2016.00082 27014019 PMC4782164

[B64] Liu Y. Piazza E. A. Simony E. Shewokis P. A. Onaral B. Hasson U.. (2017). Measuring speaker–listener neural coupling with functional near infrared spectroscopy. Scient. Rep. 7:43293. 10.1038/srep43293 28240295 PMC5327440

[B65] Maldjian J. A. Laurienti P. J. Kraft R. A. Burdette J. H. (2003). An automated method for neuroanatomic and cytoarchitectonic atlas-based interrogation of fMRI data sets. Neuroimage 19, 1233–1239. 10.1016/S1053-8119(03)00169-1 12880848

[B66] Matcher S. J. E. Cooper C. E. Cope M. Delpy D. T. (1995). Performance comparison of several published tissue near-infrared spectroscopy algorithms. Anal. Biochem. 227, 54–68. 10.1006/abio.1995.1252 7668392

[B67] McLaren D. G. Ries M. L. Xu G. Johnson S. C. (2012). A generalized form of context-dependent psychophysiological interactions. (gPPI): a comparison to standard approaches. NeuroImage 61, 1277–1286. 10.1016/j.neuroimage.2012.03.068 22484411 PMC3376181

[B68] Mermelstein P. (1975). Automatic segmentation of speech into syllabic units. J. Acoust. Soc. Am. 58, 880–883. 10.1121/1.380738 1194547

[B69] Mosconi M. W. Mack P. B. McCarthy G. Pelphrey K. A. (2005). Taking an “intentional stance” on eye-gaze shifts: a functional neuroimaging study of social perception in children. NeuroImage 27, 247–252. 10.1016/j.neuroimage.2005.03.027 16023041

[B70] Néda Z. Ravasz E. Brechet Y. Vicsek T. Barabási A. L. (2000). The sound of many hands clapping. Nature 403, 849–850. 10.1038/35002660 10706271

[B71] Noah J. A. Zhang X. Dravida S. Ono Y. Naples A. McPartland J. C. Hirsch J. (2020). Real-time eye-to-eye contact is associated with cross-brain neural coupling in angular gyrus. Front. Hum. Neurosci. 14:19. 10.3389/fnhum.2020.00019 32116606 PMC7016046

[B72] Ogawa S. Lee T. M. Kay A. R. Tank D. W. (1990). Brain magnetic resonance imaging with contrast dependent on blood oxygenation. Proceed. Natl. Acad. Sci. U.S.A. 87, 9868–9872. 10.1073/pnas.87.24.9868 2124706 PMC55275

[B73] Okamoto M. Dan I. (2005). Automated cortical projection of head-surface locations for transcranial functional brain mapping. NeuroImage 26, 18–28. 10.1016/j.neuroimage.2005.01.018 15862201

[B74] Oldfield R. C. (1971). The assessment and analysis of handedness: the edinburgh inventory. Neuropsychologia 9, 97–113. 10.1016/0028-3932(71)90067-4 5146491

[B75] Osaka N. Minamoto T. Yaoi K. Azuma M. Osaka M. (2014). Neural synchronization during cooperated humming: a hyperscanning study using fNIRS. Proc. Soc. Behav. Sci. 126, 241–243. 10.1016/j.sbspro.2014.02.395

[B76] Osaka N. Minamoto T. Yaoi K. Azuma M. Shimada Y. M. Osaka M. (2015). How two brains make one synchronized mind in the inferior frontal cortex: fNIRS-based hyperscanning during cooperative singing. Front. Psychol. 6, 1–11. 10.3389/fpsyg.2015.01811 26635703 PMC4659897

[B77] Pardo J. S. (2006). On phonetic convergence during conversational interaction. J. Acoust. Soc. Am. 119, 2382–2393. 10.1121/1.2178720 16642851

[B78] Pelphrey K. A. Morris J. P. McCarthy G. (2005). Neural basis of eye gaze processing deficits in autism. Brain 128, 1038–1048. 10.1093/brain/awh404 15758039

[B79] Pickering M. J. Garrod S. (2004). Toward a mechanistic psychology of dialogue. Behav. Brain Sci. 27, 169–190. 10.1017/S0140525X04000056 15595235

[B80] Pinti P. Aichelburg C. Gilbert S. Hamilton A. Hirsch J. Burgess P.. (2018). A review on the use of wearable functional near-infrared spectroscopy in naturalistic environments. Jap. Psychol. Res. 60, 347–373. 10.1111/jpr.12206 30643322 PMC6329605

[B81] Pinti P. Aichelburg C. Lind F. Power S. Swingler E. Merla A.. (2015). Using fiberless, wearable fNIRS to monitor brain activity in real-world cognitive tasks. J. Visual. Exp. 106:e53336. 10.3791/53336 26651025 PMC4692764

[B82] Pinti P. Tachtsidis I. Hamilton A. Hirsch J. Aichelburg C. Gilbert S.. (2020). The present and future use of functional near-infrared spectroscopy. (fNIRS). for cognitive neuroscience. Ann. NY. Acad. Sci. 1464:5. 10.1111/nyas.13948 30085354 PMC6367070

[B83] Pitcher D. Walsh V. Duchaine B. (2011). The role of the occipital face area in the cortical face perception network. Exp. Brain Res. 209, 481–493. 10.1007/s00221-011-2579-1 21318346

[B84] Piva M. Zhang X. Noah A. Chang S. W. Hirsch J. (2017). Distributed neural activity patterns during human-to-human competition. Front. Hum. Neurosci. 11:571. 10.3389/fnhum.2017.00571 29218005 PMC5703701

[B85] Poeppel D. (2014). The neuroanatomic and neurophysiological infrastructure for speech and language. Curr. Opin. Neurobiol. 28, 142–149. 10.1016/j.conb.2014.07.005 25064048 PMC4177440

[B86] Price C. J. (2012). A review and synthesis of the first 20 years of PET and fMRI studies of heard speech, spoken language, and reading. NeuroImage 62, 816–847. 10.1016/j.neuroimage.2012.04.062 22584224 PMC3398395

[B87] R Core Team (2018). R: A Language and Environment for Statistical Computing (Version 3.5.0). Vienna: R Foundation for Statistical Computing. Available online at: https://www.R-project.org

[B88] Redcay E. Schilbach L. (2019). Using second-person neuroscience to elucidate the mechanisms of social interaction. Nat. Rev. Neurosci. 20, 495–505. 10.1038/s41583-019-0179-4 31138910 PMC6997943

[B89] Richardson D. C. Dale R. (2005). Looking to understand: the coupling between speakers' and listeners' eye movements and its relationship to discourse comprehension. Cogn. Sci. 29, 1045–1060. 10.1207/s15516709cog0000_29 21702802

[B90] Rojiani R. Zhang X. Noah A. Hirsch J. (2018). Communication of emotion via drumming: dual-brain imaging with functional near-infrared spectroscopy. Soc. Cogn. Affect. Neurosci. 13, 1047–1057. 10.1093/scan/nsy076 30215809 PMC6204489

[B91] Saito D. N. Tanabe H. C. Izuma K. Hayashi M. J. Morito Y. Komeda H.. (2010). “Stay tuned”: inter-individual neural synchronization during mutual gaze and joint attention. Front. Integr. Neurosci. 4:127. 10.3389/fnint.2010.00127 21119770 PMC2990457

[B92] Sato W. Kochiyama T. Uono S. Toichi M. (2016). Neural mechanisms underlying conscious and unconscious attentional shifts triggered by eye gaze. NeuroImage 124, 118–126. 10.1016/j.neuroimage.2015.08.061 26343316

[B93] Saxe R. Kanwisher N. (2003). People thinking about thinking people. The role of the temporo-parietal junction in “theory of mind”. Neuroimage. 19, 1835–1842. 10.1016/S1053-8119(03)00230-1 12948738

[B94] Saxe R. Powell L. J. (2006). It's the Thought That Counts: Specific brain regions for one component of theory of mind. Psychol Sci. 17, 692–699. 10.1111/j.1467-9280.2006.01768.x 16913952

[B95] Schegloff E. Jefferson G. Sacks H. (1974). A simplest systematics for the organization of turn-taking for conversation. Language 50, 696–735. 10.1353/lan.1974.0010

[B96] Schilbach L. (2010). A second-person approach to other minds. Nat. Rev. Neurosci. 11:449 10.1038/nrn2805-c1 20485366

[B97] Schilbach L. (2014). On the relationship of online and offline social cognition. Front. Hum. Neurosci. 8:278. 10.3389/fnhum.2014.00278 24834045 PMC4018539

[B98] Schilbach L. Timmermans B. Reddy V. Costall A. Bente G. Schlicht T.. (2013). Toward a second-person neuroscience. Behav. Brain Sci. 36, 393–414. 10.1017/S0140525X12000660 23883742

[B99] Schippers M. Roebroeck A. Renken R. Nanetti L. Keysers C. (2010). Mapping the information flow from one brain to another during gestural communication. Proc. Natl. Acad. Sci. U.S.A. 107, 9388–9393. 10.1073/pnas.1001791107 20439736 PMC2889063

[B100] Scholkmann F. Gerber U. Wolf M. Wolf U. (2013b). End-tidal CO_2_: an important parameter for a correct interpretation in functional brain studies using speech tasks. Neuroimage 66, 71–79. 10.1016/j.neuroimage.2012.10.025 23099101

[B101] Scholkmann F. Holper L. Wolf U. Wolf M. (2013a). A new methodical approach in neuroscience: assessing inter-personal brain coupling using functional near-infrared imaging. (fNIRI). hyperscanning. Front. Hum. Neurosci. 7:813. 10.3389/fnhum.2013.00813 24348362 PMC3841755

[B102] Scholkmann F. Wolf M. Wolf U. (2013c). “The effect of inner speech on arterial CO_2_ and cerebral hemodynamics and oxygenation: a functional NIRS study,” in Oxygen Transport to Tissue XXXV. Advances in Experimental Medicine and Biology, Vol. 789, eds S. Van Huffel, G. Naulaers, A. Caicedo, D. F. Bruley, and D. K. Harrison (New York, NY: Springer New York), 81–87. 10.1007/978-1-4614-7411-1_12 23852480

[B103] Sorger B. Goebel R. Schiltz C. Rossion B. (2007). Understanding the functional neuroanatomy of acquired prosopagnosia. NeuroImage 35, 836–852. 10.1016/j.neuroimage.2006.09.051 17303440

[B104] Stephens G. J. Silbert L. J. Hasson U. (2010). Speaker–listener neural coupling underlies successful communication. Proc. Natl. Acad. Sci. U.S.A. 107, 14425–14430. 10.1073/pnas.1008662107 20660768 PMC2922522

[B105] Stivers T. Enfield N. J. Brown P. Englert C. Hayashi M. Heinemann T.. (2009). Universals and cultural variation in turn-taking in conversation. Proc. Natl. Acad. Sci. U.S.A. 106, 10587–10592. 10.1073/pnas.0903616106 19553212 PMC2705608

[B106] Strangman G. Culver J. P. Thompson J. H. Boas D. A. (2002). A quantitative comparison of simultaneous BOLD fMRI and NIRS recordings during functional brain activation. NeuroImage 17, 719–731. 10.1006/nimg.2002.1227 12377147

[B107] Tachtsidis I. Scholkmann F. (2016). False positives and false negatives in functional near-infrared spectroscopy: Issues, challenges, and the way forward. Neurophotonics 3:031405 10.1117/1.NPh.3.3.031405 27054143 PMC4791590

[B108] Tanabe H. C. Kosaka H. Saito D. N. Koike T. Hayashi M. J. Izuma K.. (2012). Hard to “tune in”: neural mechanisms of live face-to-face interaction with high-functioning autistic spectrum disorder. Front. Hum. Neurosci. 6:268. 10.3389/fnhum.2012.00268 23060772 PMC3459004

[B109] Tang H. Mai X. Wang S. Zhu C. Krueger F. Liu C. (2016). Interpersonal brain synchronization in the right temporo-parietal junction during face-to-face economic exchange. Soc. Cogn. Affect. Neurosci. 11, 23–32. 10.1093/scan/nsv092 26211014 PMC4692317

[B110] Torrence C. Compo G. P. (1998). A practical guide to wavelet analysis. B. Am. Meteorol. Soc. 79, 61–78. 10.1175/1520-0477(1998)079<0061:APGTWA>2.0.CO;2

[B111] Vanutelli M. E. Gatti L. Angioletti L. Balconi M. (2017). Affective synchrony and autonomic coupling during cooperation: a hyperscanning study. Biomed. Res. Int. 2017:3104564. 10.1155/2017/3104564 29279845 PMC5723953

[B112] Villringer A. Chance B. (1997). Non-invasive optical spectroscopy and imaging of human brain function. Trends Neurosci. 20, 435–442. 10.1016/S0166-2236(97)01132-6 9347608

[B113] Wheatley T. Boncz A. Toni I. Stolk A. (2019). Beyond the isolated brain: the promise and challenge of interacting minds. Neuron 103, 186–188. 10.1016/j.neuron.2019.05.009 31319048 PMC7789915

[B114] Williams C. E. Stevens K. N. (1972). Emotions and speech: some acoustical correlates. J. Acoust. Soc. Am. 52, 1238–1250. 10.1121/1.1913238 4638039

[B115] Wilson M. Wilson T. P. (2005). An oscillator model of the timing of turn-taking. Psych. Bull. Rev. 12, 957–968. 10.3758/BF03206432 16615316

[B116] Wrede B. Shriberg E. (2003). “Spotting “hot spots” in meetings: Human judgments and prosodic cues,” in Eighth European Conference on Speech Communication and Technology (Geneva), 2805–2808. Available online at: https://www.isca-speech.org/archive/eurospeech_2003/e03_2805.html

[B117] Ye J. C. Tak S. Jang K. E. Jung J. Jang J. (2009). NIRS-SPM: statistical parametric mapping for near-infrared spectroscopy. NeuroImage 44, 428–447. 10.1016/j.neuroimage.2008.08.036 18848897

[B118] Zhang W. Yartsev M. M. (2019). Correlated neural activity across the brains of socially interacting bats. Cell 178, 413–428. 10.1016/j.cell.2019.05.023 31230710 PMC6625887

[B119] Zhang X. Noah J. A. Dravida S. Hirsch J. (2017). Signal processing of functional NIRS data acquired during overt speaking. Neurophotonics 4:041409. 10.1117/1.NPh.4.4.041409 28924564 PMC5592780

[B120] Zhang X. Noah J. A. Dravida S. Hirsch J. (2020). Optimization of wavelet coherence analysis as a measure of neural synchrony during hyperscanning using functional near-infrared spectroscopy. Neurophotonics 7:015010. 10.1117/1.NPh.7.1.015010 32206677 PMC7047008

[B121] Zhang X. Noah J. A. Hirsch J. (2016). Separation of the global and local components in functional near-infrared spectroscopy signals using principal component spatial filtering. Neurophotonics 3:015004. 10.1117/1.NPh.3.1.015004 26866047 PMC4742567

[B122] Zivotofsky A. Z. Hausdorff J. M. (2007). The sensory feedback mechanisms enabling couples to walk synchronously: an initial investigation. J. Neuroeng. Rehab. 4, 1–5. 10.1186/1743-0003-4-28 17686150 PMC1973071

