# Supplementary material for: Interpersonal Agreement and Disagreement During Face-to-Face Dialogue: An fNIRS Investigation
Source: Front Hum Neurosci. 2021 Jan 13;14:606397. doi: 10.3389/fnhum.2020.606397 (PMC7874076; doi:10.3389/fnhum.2020.606397)
Supplement: Supplementary file 1 [file Table_1.DOCX]

Supplementary Material

**Table S1.** Group median coordinates, anatomical regions, and atlas-based probabilities for each channel. Montreal Neurological Institute (MNI) coordinates for each of the 42 channels per participant were determined by digitizing the locations of the optodes in relation to the 10-20 system based on conventional landmarks. See Figure 1B in main text.

| **Table S1. Channels, group median coordinates, anatomical regions, and atlas-based probabilities** | | | | | | |
| --- | --- | --- | --- | --- | --- | --- |
| Channel | MNI Coordinates^1^ | | | Anatomical Region | BA^2^ | Probability |
| number | X | Y | Z |  |  |  |
|  |  |  |  |  |  |  |
| 1 | 62 | -21 | 51 | Primary Somatosensory Cortex | 1 | 0.288 |
|  |  |  |  | Primary Somatosensory Cortex | 2 | 0.273 |
|  |  |  |  | Primary Somatosensory Cortex | 3 | 0.270 |
|  |  |  |  | Primary Motor Cortex | 4 | 0.169 |
|  |  |  |  |  |  |  |
| 2 | 68 | -35 | 40 | Supramarginal Gyrus | 40 | 0.890 |
|  |  |  |  | Primary Somatosensory Cortex | 2 | 0.110 |
|  |  |  |  |  |  |  |
| 3 | 66 | -9 | 39 | Primary Motor Cortex | 4 | 0.844 |
|  |  |  |  | Primary Somatosensory Cortex | 3 | 0.156 |
|  |  |  |  |  |  |  |
| 4 | 21 | 46 | 48 | Dorsolateral Prefrontal Cortex | 9 | 0.828 |
|  |  |  |  | Frontal Eye Fields | 8 | 0.172 |
|  |  |  |  |  |  |  |
| 5 | 56 | -74 | 20 | Angular Gyrus | 39 | 0.759 |
|  |  |  |  | Extrastriate Visual Cortex (V3) | 19 | 0.241 |
|  |  |  |  |  |  |  |
| 6 | 68 | -49 | 21 | Superior Temporal Gyrus | 22 | 0.687 |
|  |  |  |  | Supramarginal Gyrus | 40 | 0.313 |
|  |  |  |  |  |  |  |
| 7 | 70 | -24 | 28 | Supramarginal Gyrus | 40 | 0.602 |
|  |  |  |  | Primary Somatosensory Cortex | 2 | 0.275 |
|  |  |  |  | Primary Somatosensory Cortex | 1 | 0.123 |
|  |  |  |  |  |  |  |
| 8 | 67 | 1 | 24 | Pre- and Supplementary Motor Cortex | 6 | 0.683 |
|  |  |  |  | Primary Motor Cortex | 4 | 0.317 |
|  |  |  |  |  |  |  |
| 9 | 60 | 27 | 22 | Pars Triangularis | 45 | 0.557 |
|  |  |  |  | Dorsolateral Prefrontal Cortex | 46 | 0.443 |
|  |  |  |  |  |  |  |
| 10 | 40 | 49 | 33 | Dorsolateral Prefrontal Cortex | 9 | 0.545 |
|  |  |  |  | Dorsolateral Prefrontal Cortex | 46 | 0.236 |
|  |  |  |  | Frontopolar Cortex | 10 | 0.219 |
|  |  |  |  |  |  |  |
| 11 | 64 | -61 | -1 | Occipitotemporal Cortex | 37 | 0.764 |
|  |  |  |  | Middle Temporal Gyrus | 21 | 0.236 |
|  |  |  |  |  |  |  |
| 12 | 72 | -37 | 9 | Superior Temporal Gyrus | 22 | 0.866 |
|  |  |  |  | Auditory Primary and Association Cortex | 42 | 0.134 |
|  |  |  |  |  |  |  |
| 13 | 70 | -11 | 14 | Subcentral Area | 43 | 0.561 |
|  |  |  |  | Auditory Primary and Association Cortex | 42 | 0.303 |
|  |  |  |  | Superior Temporal Gyrus | 22 | 0.135 |

| 14 | 64 | 12 | 7 | Pars Opercularis | 44 | 0.426 |
| --- | --- | --- | --- | --- | --- | --- |
|  |  |  |  | Pars Triangularis | 45 | 0.281 |
|  |  |  |  | Superior Temporal Gyrus | 22 | 0.158 |
|  |  |  |  | Pre- and Supplementary Motor Cortex | 6 | 0.135 |
|  |  |  |  |  |  |  |
| 15 | 50 | 48 | 18 | Dorsolateral Prefrontal Cortex | 46 | 0.667 |
|  |  |  |  | Frontopolar Cortex | 10 | 0.333 |
|  |  |  |  |  |  |  |
| 16 | 23 | 62 | 29 | Frontopolar Cortex | 10 | 0.842 |
|  |  |  |  | Dorsolateral Prefrontal Cortex | 9 | 0.158 |
|  |  |  |  |  |  |  |
| 17 | 68 | -49 | -9 | Occipitotemporal Cortex | 37 | 0.492 |
|  |  |  |  | Middle Temporal Gyrus | 21 | 0.429 |
|  |  |  |  | Inferior Temporal Gyrus | 20 | 0.080 |
|  |  |  |  |  |  |  |
| 18 | 73 | -25 | -4 | Middle Temporal Gyrus | 21 | 0.699 |
|  |  |  |  | Superior Temporal Gyrus | 22 | 0.301 |
|  |  |  |  |  |  |  |
| 19 | 68 | -4 | -8 | Middle Temporal Gyrus | 21 | 0.582 |
|  |  |  |  | Superior Temporal Gyrus | 22 | 0.418 |
|  |  |  |  |  |  |  |
| 20 | 56 | 38 | 1 | Pars Triangularis | 45 | 0.555 |
|  |  |  |  | Inferior Frontal Gyrus | 47 | 0.445 |
|  |  |  |  |  |  |  |
| 21 | 36 | 63 | 15 | Frontopolar Cortex | 10 | 1.000 |
|  |  |  |  |  |  |  |
| 22 | -61 | -21 | 50 | Primary Somatosensory Cortex | 2 | 0.306 |
|  |  |  |  | Primary Somatosensory Cortex | 1 | 0.273 |
|  |  |  |  | Primary Somatosensory Cortex | 3 | 0.255 |
|  |  |  |  | Primary Motor Cortex | 4 | 0.166 |
|  |  |  |  |  |  |  |
| 23 | -20 | 48 | 47 | Dorsolateral Prefrontal Cortex | 9 | 0.678 |
|  |  |  |  | Frontal Eye Fields | 8 | 0.322 |
|  |  |  |  |  |  |  |
| 24 | -64 | -10 | 38 | Primary Motor Cortex | 4 | 0.502 |
|  |  |  |  | Pre- and Supplementary Motor Cortex | 6 | 0.376 |
|  |  |  |  | Primary Somatosensory Cortex | 3 | 0.122 |
|  |  |  |  |  |  |  |
| 25 | -66 | -35 | 40 | Supramarginal Gyrus | 40 | 0.873 |
|  |  |  |  | Primary Somatosensory Cortex | 2 | 0.127 |
|  |  |  |  |  |  |  |
| 26 | -38 | 48 | 31 | Dorsolateral Prefrontal Cortex | 9 | 0.434 |
|  |  |  |  | Dorsolateral Prefrontal Cortex | 46 | 0.333 |
|  |  |  |  | Frontopolar Cortex | 10 | 0.233 |
|  |  |  |  |  |  |  |
| 27 | -57 | 25 | 19 | Pars Triangularis | 45 | 0.678 |
|  |  |  |  | Dorsolateral Prefrontal Cortex | 46 | 0.322 |
|  |  |  |  |  |  |  |
| 28 | -65 | -1 | 23 | Pre- and Supplementary Motor Cortex | 6 | 0.655 |
|  |  |  |  | Primary Motor Cortex | 4 | 0.196 |
|  |  |  |  | Subcentral Area | 43 | 0.149 |

| 29 | -68 | -24 | 28 | Supramarginal Gyrus | 40 | 0.607 |
| --- | --- | --- | --- | --- | --- | --- |
|  |  |  |  | Primary Somatosensory Cortex | 2 | 0.245 |
|  |  |  |  | Primary Somatosensory Cortex | 1 | 0.147 |
|  |  |  |  |  |  |  |
| 30 | -67 | -49 | 20 | Superior Temporal Gyrus | 22 | 0.631 |
|  |  |  |  | Supramarginal Gyrus | 40 | 0.266 |
|  |  |  |  | Angular Gyrus | 39 | 0.103 |
|  |  |  |  |  |  |  |
| 31 | -56 | -71 | 22 | Angular Gyrus | 39 | 0.724 |
|  |  |  |  | Extrastriate Visual Cortex (V3) | 19 | 0.276 |
|  |  |  |  |  |  |  |
| 32 | -21 | 62 | 29 | Frontopolar Cortex | 10 | 0.760 |
|  |  |  |  | Dorsolateral Prefrontal Cortex | 9 | 0.240 |
|  |  |  |  |  |  |  |
| 33 | -48 | 46 | 15 | Dorsolateral Prefrontal Cortex | 46 | 0.799 |
|  |  |  |  | Frontopolar Cortex | 10 | 0.201 |
|  |  |  |  |  |  |  |
| 34 | -62 | 9 | 5 | Pars Opercularis | 44 | 0.550 |
|  |  |  |  | Pre- and Supplementary Motor Cortex | 6 | 0.332 |
|  |  |  |  | Pars Triangularis | 45 | 0.118 |
|  |  |  |  |  |  |  |
| 35 | -68 | -13 | 16 | Subcentral Area | 43 | 0.717 |
|  |  |  |  | Primary Somatosensory Cortex | 1 | 0.181 |
|  |  |  |  | Auditory Primary and Association Cortex | 42 | 0.102 |
|  |  |  |  |  |  |  |
| 36 | -70 | -39 | 11 | Superior Temporal Gyrus | 22 | 0.911 |
|  |  |  |  | Middle Temporal Gyrus | 21 | 0.089 |
|  |  |  |  |  |  |  |
| 37 | -64 | -61 | 2 | Occipitotemporal Cortex | 37 | 0.537 |
|  |  |  |  | Middle Temporal Gyrus | 21 | 0.463 |
|  |  |  |  |  |  |  |
| 38 | -34 | 62 | 15 | Frontopolar Cortex | 10 | 1.000 |
|  |  |  |  |  |  |  |
| 39 | -54 | 37 | -1 | Inferior Frontal Gyrus | 47 | 0.604 |
|  |  |  |  | Pars Triangularis | 45 | 0.396 |
|  |  |  |  |  |  |  |
| 40 | -67 | -6 | -11 | Middle Temporal Gyrus | 21 | 0.571 |
|  |  |  |  | Superior Temporal Gyrus | 22 | 0.429 |
|  |  |  |  |  |  |  |
| 41 | -71 | -27 | -2 | Middle Temporal Gyrus | 21 | 0.701 |
|  |  |  |  | Superior Temporal Gyrus | 22 | 0.179 |
|  |  |  |  | Auditory Primary and Association Cortex | 42 | 0.120 |
|  |  |  |  |  |  |  |
| 42 | -68 | -50 | -7 | Occipitotemporal Cortex | 37 | 0.652 |
|  |  |  |  | Middle Temporal Gyrus | 21 | 0.348 |

^1^Coordinates are based on the MNI system and (-) indicates left hemisphere. ^2^BA = Brodmann Area identified by the TD ICBM MNI atlas (Maldjian et al., 2003)
